# Resting state functional connectivity predictors of treatment response to electroconvulsive therapy in depression

**DOI:** 10.1038/s41598-019-41175-4

**Published:** 2019-03-25

**Authors:** M. Moreno-Ortega, J. Prudic, S. Rowny, G. H. Patel, A. Kangarlu, S. Lee, J. Grinband, T. Palomo, T. Perera, M. F. Glasser, D. C. Javitt

**Affiliations:** 1 Division of Experimental Therapeutics, Department of Psychiatry, New York State Psychiatric Institute/Columbia University Medical Center, New York, NY USA; 2 Centro de Investigacion Biomedica en Red de Salud Mental (CIBERSAM), Madrid, Spain; 3 Department of Psychiatry and Biostatistics, New York State Psychiatric Institute/Columbia University, New York, NY USA; 4 Department of Psychiatry, Radiology and Biomedical Engineering, Columbia University, New York, NY USA; 5 Department of Psychiatry, Complutense University, Madrid, Spain; 6 Departments of Radiology and Neuroscience, Washington University Medical School, St. Louis, MO USA

## Abstract

There is increasing focus on use of resting-state functional connectivity (RSFC) analyses to subtype depression and to predict treatment response. To date, identification of RSFC patterns associated with response to electroconvulsive therapy (ECT) remain limited, and focused on interactions between dorsal prefrontal and regions of the limbic or default-mode networks. Deficits in visual processing are reported in depression, however, RSFC with or within the visual network have not been explored in recent models of depression. Here, we support prior studies showing in a sample of 18 patients with depression that connectivity between dorsal prefrontal and regions of the limbic and default-mode networks serves as a significant predictor. In addition, however, we demonstrate that including visual connectivity measures greatly increases predictive power of the RSFC algorithm (>80% accuracy of remission). These exploratory results encourage further investigation into visual dysfunction in depression, and use of RSFC algorithms incorporating the visual network in prediction of response to both ECT and transcranial magnetic stimulation (TMS), offering a new framework for the development of RSFC-guided TMS interventions in depression.

## Introduction

Major depressive disorder (MDD) is a severe mental disorder that affects up to 20% of the population worldwide. Approximately 50% of individuals with MDD fail to respond adequately to anti-depressant medications. For individuals with treatment resistant depression (TRD) electroconvulsive therapy (ECT) is the most effective treatment. However, ECT requires general anesthesia and may be accompanied by adverse cognitive effects, reducing its tolerability.

The response and remission rates for patients with depression without psychotic symptoms are 70% and 50%, respectively^1^. As a result, a significant number of individuals may be exposed to the significant risks of ECT without tangible benefit. At present, there are no measures available that are capable of differentiating responders from non-responders, and thus from preventing unneeded treatments. Also, despite considerable research, neural mechanisms underlying ECT effectiveness remain largely unknown, inhibiting the search for safer or more effective alternatives.

Prominent theories of both TRD and ECT response emphasize disruptions of resting state functional connectivity (RSFC) between large-scale brain networks in TRD that may be reversed with ECT treatment. To date, such studies have focused most prominently on interactions between regions of the fronto-parietal network especially dorsolateral prefrontal cortex (DLPFC; BA46, BA9 and BA8) and limbic regions such as subgenual anterior cingulate cortex (sgACC; BA25) or amygdala, and on fronto-limbic dysconnectivity. Other RSFC networks^2^, such as default-mode (DMN) and visual (VIS), have been investigated to a more limited extent.

The role of sgACC in TRD was first proposed ~20 years ago^3–5^ based on volumetric analysis, cerebral blood flow (CBF) and glucose metabolism. These findings complemented prior results with DLPFC^6–8^. Subsequent studies suggested an additional role for fronto-limbic dysregulation in both depressive illness and healthy sadness^9–11^. These theories led to development of experimental treatments such as deep brain stimulation (DBS) of the sgACC for treatment of persistent depressive symptoms^11–14^. In addition, non-invasive brain stimulation approaches such as transcranial magnetic stimulation (TMS) may be effective only to the degree that they effectively target regions of DLPFC that are anti-correlated with sgACC^15^.

Successive studies have investigated sgACC connectivity and fronto-limbic balance in relationship to treatment response to antidepressant treatments, including ECT^16–20^. For example, in one recent study, increased fractional amplitude of low frequency fluctuation (fALFF) involving sgACC significantly predicted ECT response and declined over time during ECT treatment. Functional connectivity between sgACC and additional limbic regions were also significantly reduced over the course of treatment. Nevertheless, the degree of variance explained by sgACC fALFF (~25%) was relatively modest and correlations between reduced fALFF and symptoms were not significant^21^. Changes in RSFC within dorsal ACC, medial prefrontal (MPFC) and lateral parietal cortex^22^, and between sgACC and DLPFC^23^ have also been associated with treatment improvement following ECT, but baseline patterns have not been shown to predict response.

Disturbances in other networks, such as the DMN and VIS, have also been reported in depression and ECT response, but have been less studied^24^. DMN is activated by internally focused cognitive processes such as mind wandering, self-reference, remembering the past and planning the future^25–27^. Lack of DMN suppression has been reported in depression during performance of cognitive demanding tasks^28–31^, and at rest^32–35^. DMN contains discrete anterior and posterior nodes in rostral MPFC and posterior cingulate (PCC) cortex, respectively^36^. Dissociation within the DMN^33,37^ and anterior-to-posterior hyperconnectivity^38^ have also been associated with depression.

The VIS cortex plays an important role in facial perception and expression as well as in emotion processing^39,40^. Processing of facial expressions has a predictive power to discriminate MDD patients from healthy controls^41–43^, and identifying patients who will respond to antidepressants^44^. Increased BOLD responses to sad stimuli in right sgACC and VIS cortex may be predictive of clinical recovery^45^, and respond to antidepressant treatment^46–48^. Deficits in VIS activation^49,50^ and processing of neutral facial^51^ expressions have also been reported in depression.

VIS perception, including functions such as contrast sensitivity, are altered in patients with depression, and normalized with remission after antidepressive therapy^52–54^. Alterations in VIS association (higher order) regions during perception, attention, working memory and VIS categorization may also occur in MDD^55–57^. Deficits are observed even in tasks involving simple VIS stimuli with no demand on emotion processing^49,50^, suggesting a basic dysfunction on processing information.

In this exploratory study, we evaluate RSFC pattern prior to and following ECT treatment, and evaluate both predictors and clinical correlates of response. RSFC patterns between DLPFC and both DMN and VIS networks, and between DMN and VIS as well as RSFC within these networks (“network homogeneity”) were computed. Based upon the accumulating data regarding VIS dysfunction in MDD, we hypothesized that including VIS connectivity along with that of more extensively studied fronto-limbic and DMN networks would significantly enhance predictive value of network models.

## Results

Mean symptom reduction following ECT was 62% (62.2 ± 5.9). Further, 9 patients out of 18 (50%) were classified as remitters and 9 patients out of 18 (50%) as non-remitters. After treatment, average HDRS-24 in remitter (4.7 ± 0.8) differed from that of non-remitter (15.2 ± 1.3; p < 0.000). No significant differences in sex (60% female vs. 60% female), age (53.2 ± 3.7 vs. 50.2 ± 4.5) or HDRS-24 baseline scores (25.8 ± 1.2 vs. 27.2 ± 1.4) were found between remitters and non-remitters.

### Response prediction

Table 1 summarizes findings regarding pretreatment RSFC patterns within and between DLPFC, DMN and VIS networks correlated to treatment response, with p-values greater than 0.05. Reduced connectivity (less anticorrelated) between DLPFC and DMN, or between DMN and VIS, and reduced connectivity (less correlated) between DLPFC and VIS, or within DLPFC, DMN or VIS, were associated with improvement in depression scores. After multiple comparison correction, decreased connectivity within aDMN(10r), between DLPFC(46) and aDMN(s32), or between DLPFC(p9-46v) and VIS(MT+), remained significant. Even if decreased connectivity within VIS(ventral) did not survive multiple comparison correction, it added predictive value to final models. The main model involved pretreatment RSFC within aDMN(10r) and VIS(ventral), with 100% accuracy of remission within this sample. Leave-one-out cross validation (LOOCV) on FDR corrected models, adjusted by motion displacement regressors, showed 0.83–0.89 prediction accuracy.

**Table 1:** Connectivity measures at baseline and change in depression scores.

Connectivity at baseline		Correlation analyses			Partial correlation analyses					
					Adjusted by RelRMS			Adjusted by AbsRMS		
		r	uncorrected p-value	corrected p-value	r	uncorrected p-value	corrected p-value	r	uncorrected p-value	corrected p-value
**Initial DLPFC**_**neg**_ **analyses**	DLPFC(46)-sgACC(25)	0.535	0.022	0.074	0.521	0.032	0.086	0.555	0.021	0.083
	DLPFC(46)-aDMN(a24)	0.576	0.012	0.074	0.564	0.018	0.086	0.576	0.015	0.083
	DLPFC(p9-46v)-aDMN(a24)	0.517	0.028	0.074	0.529	0.029	0.086	0.523	0.031	0.083
**Subsequent network analyses**	**DLPFC(46)-aDMN(s32)**	**0.685**	**0.002**	**0.033**	**0.679**	**0.003**	**0.040**	**0.684**	**0.002**	**0.047**
	**DLPFC(p9-46v)-VIS(MT+)**	−0.610	0.007	0.091	−**0.672**	**0.003**	**0.040**	−**0.663**	**0.004**	**0.047**
	aDMN(10r)-VIS(MT+)	0.490	0.039	0.164	0.475	0.054	0.171	0.496	0.043	0.181
	Intra-DLPFC(46)	−0.500	0.035	0.164	−0.497	0.043	0.171	−0.500	0.041	0.181
	Intra-DLPFC(p9-46v/46)	−0.490	0.039	0.164	−0.477	0.053	0.171	−0.524	0.031	0.181
	**Intra-aDMN(10r)**	−**0.699**	**0.001**	**0.033**	−**0.694**	**0.002**	**0.040**	−**0.709**	**0.001**	**0.047**
	Intra-aDMN(s32/10r)	−0.550	0.018	0.164	−0.537	0.026	0.171	−0.550	0.022	0.181
	Intra-VIS(ventral)	−0.523	0.026	0.164	−0.531	0.028	0.171	−0.521	0.032	0.181
***Follow-up VIS analyses***	*DLPFC(p9-46v)-MT*+ *(LO*1*)*	−*0.606*	*0.008*	*0.060*	−*0.593*	*0.01*2	*0.065*	−*0.609*	*0.0*1*0*	*0.071*
	***DLPFC(p9-46v)- MT***+ ***(FST)***	−***0.661***	***0.003***	***0.045***	−***0.745***	***0.001***	***0.010***	−***0.740***	***0.001***	***0.011***
	*DLPFC(p9-46v)-MT*+ (*V3CD)*	−*0.539*	*0.0*21	*0.060*	−*0.523*	*0.03*1	*0.067*	−*0.54*1	*0.025*	*0.072*
	*Intra-ventral(FFC/ventral)*	−*0.523*	*0.026*	*0.060*	−*0.525*	*0.030*	*0.067*	−*0.522*	*0.032*	*0.072*
	*Intra-ventral(V8/ventral)*	−*0.532*	*0.023*	*0.060*	−*0.548*	*0.023*	*0.067*	−*0.531*	*0.028*	*0.072*
	*Intra-ventral(PIT/ventral)*	−*0.564*	*0.015*	*0.060*	−*0.593*	*0.012*	*0.065*	−*0.566*	*0.018*	*0.071*
	*Intra-ventral(VMV1/ventral)*	−*0.530*	*0.024*	*0.060*	−*0.516*	*0.034*	*0.067*	−*0.581*	*0.015*	*0.071*
	*Intra-ventral(VMV3/ventral)*	−*0.487*	*0.040*	*0.081*	−*0.498*	*0.042*	*0.067*	−*0.486*	*0.048*	*0.096*

The nomenclature used in this table and along the results section is based on the HCP’s multimodal parcellation, each parcel used for analyses appears in parenthesis preceded by the region to which it pertains; e.g., aDMN(10r) refers to parcels 10r within the anterior medial locus of DMN (or aDMN), DLPFC(46) refers to parcel 46 within the DLPF, etc. For visual system (or VIS) analyses, 2 levels of analyses were explored (VIS region vs. parcel within region); e.g., VIS(MT+) refers to the MT+ region within the visual system. In follow up VIS analyses, e.g., MT+ (FST) refers to the parcel FST within the MT+ region, etc. Initial DLPFC_neg_ analyses tested 8 correlations involving RSFC between DLPFC_neg_(46, p9-46v, a9-46v, 9-46d) and sgACC(25) or rostral ACC(a24) to choose significant (uncorrected p values) DLPFC_neg_ parcels for subsequent network analyses. Subsequent network analyses tested 38 correlations involving RSFC within and between DLPFC_neg_(46, p9-46v), DMN or VIS networks. Follow-up VIS analyses queried which parcels within MT+ (p = 9) and ventral (p = 7) were driving the observed regional comparisons. These analyses used all parcels within each region and were therefore FDR corrected for 16 comparisons each. We include in Table 1 all significant (uncorrected p values) connections from correlation analyses; those that survived multiple comparison correction controlling for FDR are highlighted.

### DLPFC-sgACC/rostral ACC

Consistent with previous work^15^, connectivity (reduced anticorrelation) between DLPFC(46, p9-46v) and sgACC(25) or rostral ACC(a24) were associated with treatment response but did not survive to multiple comparison correction (Supplementary Results).

### DLPFC-DMN

**Figure 1:**
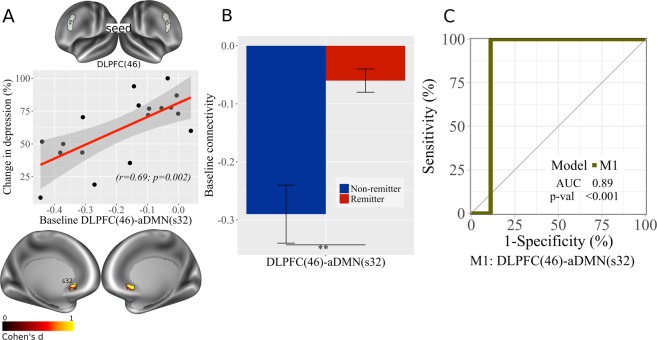
DLPFC, sgACC and rostral ACC inter-connectivity at baseline. (**A**) Correlation plot with baseline RSFC data for DLPFC(46) – aDMN(s32) associated with improvement in depression (% change in depression scores) ([pre-post/pre] × 100); brain images displayed at Cohen’s d values > 0 (Cohen’s d = 2t/sqrt(dfe)), colors represent surface vertices with positive correlation (red-yellow) between RSFC data and improvement in depression (% change in depression scores, [pre-post/pre] × 100). (**B**) Bar plot with baseline DLPFC(46) – aDMN(s32) comparison between non-remitters and remitters; bars represent connectivity values; **p < 0.01. Error bars represent standard error. (**C**) ROC curve analysis of DLPFC(46) – aDMN(s32) model. AUC, area under the curve; p-val, from Likelihood Ratio Test.

A significant (r = 0.69, p = 0.002; Fig. 1A) correlation was observed between DLPFC(46)-aDMN(s32) connectivity and treatment response, but not for DLPFC(p9-46v). Remitters showed reduced anticorrelation compared to non-remitters (Fig. 1B). A ROC curve of this model showed significant area under the curve (AUC = 0.89, p < 0.001, 95% CI: 0.67–1; Fig. 1C). No significant correlations were observed between DLPFC(46, p9-46v) and pDMN(31pv, v23ab).

### *DLPFC/DMN*-VIS

**Figure 2:**
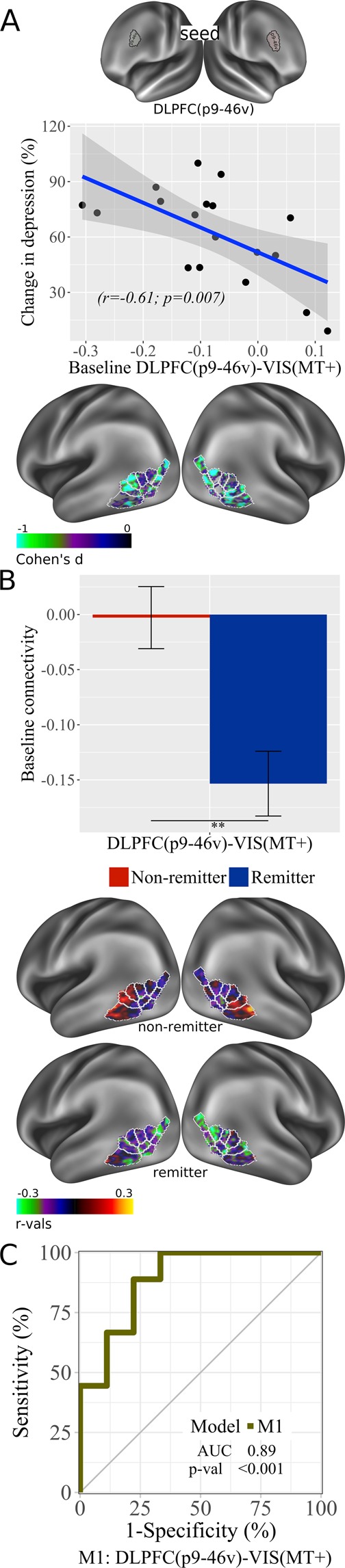
DLPFC and VIS inter-connectivity at baseline. (**A**) Correlation plot with baseline RSFC data for DLPFC(p9-46v) – VIS(MT+) associated with improvement in depression (% change in depression scores) ([pre-post/pre] × 100); brain images displayed at Cohen’s d values < 0 (Cohen’s d = 2t/sqrt(dfe)) for DLPFC(p9-46v) – VIS(MT+), colors represent surface vertices with negative (blue-violet) correlation between RSFC data and improvement in depression (% change in depression scores) ([pre-post/pre] × 100). (**B**) Bar plot with baseline DLPFC(p9-46v) – VIS(MT+) comparison between non-remitters and remitters; bars represent connectivity values, **p < 0.01. Error bars represent standard error. Brain images display RSFC between DLPFC(p9-46v) and VIS(MT+) for non-remitters and remitters; colors represent surface vertices with negative (blue-violet) correlation (−0.3≤ r ≤0.3) with VIS(MT+). (**C**) ROC curve analysis of DLPFC(p9-46v) – VIS(MT+) model. AUC, area under the curve; p-val, from Likelihood Ratio Test.

For DLPFC-VIS connectivity, a significant negative correlation was observed for baseline DLPFC(p9-46v)-VIS(MT+) (r = −0.61, p = 0.007; Fig. 2A) and treatment response, but not for DLPFC(46). Remitters showed significantly greater anti-correlation between DLPFC(p9-46v)-VIS(MT+) at baseline than non-remitters (Fig. 2B). In remitters, significant correlations with improvement were observed across multiple parcels. AUC for this model was highly predictive of remission (AUC = 0.89, p < 0.001, 95% CI: 0.70–1; Fig. 2C).

By contrast, for DMN-VIS connectivity, a significant positive correlation with treatment response was found for aDMN(10r)-VIS(MT+), but did not survive to multiple comparison correction (Supplementary Results).

### Within-network connectivity

**Figure 3:**
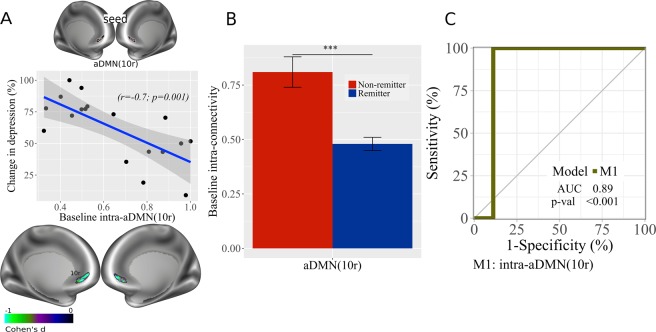
DMN intra-connectivity at baseline. (**A**) Correlation plot with baseline RSFC data for within aDMN(10r), associated with improvement in depression depression (% change in depression scores) ([pre-post/pre] × 100); brain images displayed at Cohen’s d values < 0 (Cohen’s d = 2t/sqrt(dfe)), colors represent surface vertices with negative (blue-violet) correlation between RSFC data and improvement in depression depression (% change in depression scores) ([pre-post/pre] × 100). (**B**) Bar plot with baseline RSFC within aDMN(10r) comparison between non-remitters and remitters; bars represent connectivity values; ***p < 0.001. Error bars represent standard error. (**C**) ROC curve analysis of RSFC within aDMN(10r) model. AUC, area under the curve; p-val, from Likelihood Ratio Test.

In addition to pairwise connectivity between networks, we also evaluated connectivity within networks. Reduced connectivity within aDMN(10r) (r = −0.70, p = 0.001; Fig. 3A) correlated with clinical improvement. Remitters showed significantly reduced intrinsic connectivity than non-remitters (Fig. 3B). AUC for intra-aDMN(10r) (AUC = 0.89, p < 0.001, 95% CI: 0.67–1; Fig. 3C) significantly predicted remission.

**Figure 4:**
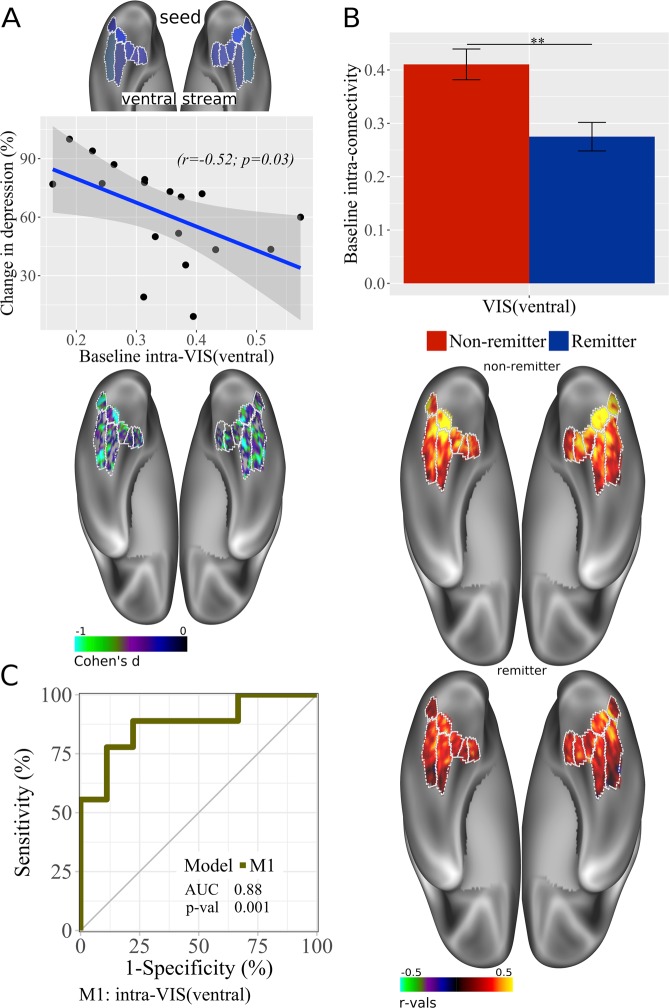
VIS intra-connectivity at baseline. (**A**) (from up-down) Correlation plots with baseline RSFC data for within VIS(ventral) associated with improvement in depression (% change in depression scores) ([pre-post/pre] × 100); brain images displayed at Cohen’s d values < 0 (Cohen’s d = 2t/sqrt(dfe)), colors represent surface vertices with negative (blue-violet) correlation between RSFC data and improvement in depression (% change in depression scores) ([pre-post/pre] × 100). (**B**) (from up-down) Bar plot with baseline RSFC within VIS(ventral) comparison between non-remitters and remitters; bars represent connectivity values; **p < 0.01. Error bars represent standard error. Brain images display RSFC within VIS(ventral) for non-remitters and remitters, colors represent surface vertices with negative (blue-violet) or positive (red-yellow) correlation (−0.5≤ r ≤0.5) within VIS(ventral). (**C**) ROC curve analyses of RSFC within VIS(ventral) model. AUC, area under the curve; p-val, from Likelihood Ratio Test.

For VIS, reduced intrinsic connectivity within the ventral stream (intra-regional connectivity) predicted treatment response (r = −0.52, p = 0.03; Fig. 4A**)**. Remitters showed less intrinsic connectivity than non-remitters (Fig. 4B**)**. The AUC for prediction of remission by intra-regional connectivity within the ventral stream was also significant (AUC = 0.88, p = 0.001, 95% CI: 0.68–1; Fig. 4C).

### Correlation between connectivity measures at baseline

**Figure 5:**
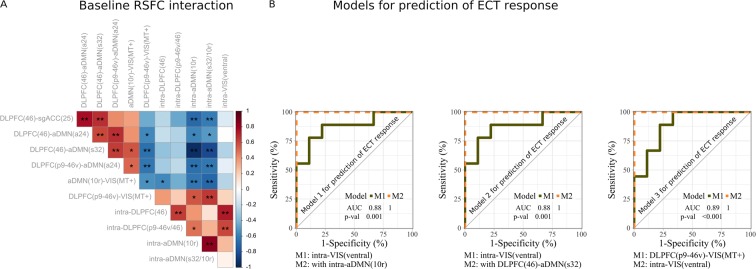
Models for prediction of ECT response. (**A**) Correlation matrix of pairwise correlation between pretreatment connections within and between DLPFC, DMN and VIS networks; colors (red, blue) represent strength of correlations (+1, −1); *<0.05, **<0.01. (B) (from left-right) ROC curve analyses of Model 1: Baseline RSFC within-VIS(ventral) and within-aDMN(10r). Model 2: Baseline RSFC within-VIS(ventral) and between DLPFC(46) – DMN(s32). Model 3: Baseline RSFC between DLPFC(p9-46v) – VIS(MT+) and within-VIS(ventral). AUC, area under the curve; p-val, from Likelihood Ratio Test.

We generated a correlation matrix of pairwise correlation between all pretreatment connections (Fig. 5A) within and between DLPFC, DMN and VIS networks. These analyses most importantly revealed positive correlations between intra-aDMN and DLPFC-VIS connectivity, or negative correlations between intra-aDMN and DLPFC-aDMN connectivity. Conversely, intra-VIS connectivity was not correlated to intra-aDMN, DLPFC-VIS or DLPFC-aDMN connectivity.

### Two Factor Models for prediction of magnitude of improvement

No significant additivity was observed for models involving only DLPFC and DMN regions. Specifically, the combination of sgACC(25) and aDMN(24) did not show significantly increased predictive ability vs. DLPFC(46) connectivity to either region alone (Supplementary Table 1).

By contrast, when either intrinsic ventral VIS connectivity or pairwise connectivity between VIS regions and DLPFC or DMN were included, significant additive predictive values were obtained. Thus, the combination of intrinsic ventral VIS connectivity with either intrinsic aDMN(10r) connectivity (Table 2, Model 1), pairwise connectivity between DLPFC(46)-aDMN(s32) (Table 2, Model 2) or DLPFC(p9-46v)-VIS(MT+) (Table 2, Model 3), all resulted in significant improvements vs. the corresponding one-predictor models (R^2^ change ≥ 0.3, p < 0.05). Each of these models accounted for ~60% of the variance in treatment response.

**Table 2:** Multiple linear regression Models for prediction of change in depression following ECT, based upon pre-ECT connectivity patterns.

Linear regression^a^		Model 1^b^				Model 2^c^				Model 3^d^			
		Coefficients		Change statistics		Coefficients		Change statistics		Coefficients		Change statistics	
		Partial^e^	p-val	R^2^	p(F)	Partial^e^	p-val	R^2^	p(F)	Partial^e^	p-val	R^2^	p(F)
***Step***_***1***_	DLPFC(p9-46v)-VIS(MT+)									−0.62	0.008	0.37	0.007
	Intra-VIS(ventral)	−0.49	0.048	0.27	0.026	−0.49	0.046	0.27	0.026	−0.53	0.03	0.18	0.029
***Step***_***2***_	Intra-aDMN(10r)	−0.69	0.003	0.34	0.003								
	DLPFC(46)-aDMN(s32)					0.67	0.003	0.32	0.003				
	DLPFC(p9-46v)-VIS(MT+)												
R^2^	adj. R^2^	0.61		0.56		0.60		0.54		0.55		0.49	

^a^Stepwise method. Step_1_: 1-factor; Step_2_: 2-factor.

^b^Model 1: Intra-VIS ventral and intra-aDMN(10r).

^c^Model 2: Intra-VIS ventral and DLPFC(46)-aDMN(s32).

^d^Model 3: DLPFC(p9-46v)-VIS(MT+) and intra-VIS ventral.

^e^Partial correlation coefficients: independent contribution of each factor after adjusting by the influence of the second factor in Models 1–4.

### Two factor models for prediction of remission

Logistic regression analyses and LOOCV were used in order to assess the degree to which the models shown above predicted remission. Intrinsic ventral VIS connectivity alone predicted 77.8% of remitters and non-remitters. The addition of intrinsic aDMN(10r) connectivity in Model 1 (Table 3), increased predictive value to 100% accuracy for both remitters and non-remitters. In Model 2 (Table 3), the addition of DLPFC(46)-DMN(s32) connectivity, also improved predictive value, with 94% accuracy of remission.

**Table 3:** Logistic regression of Models for prediction of ECT response.

Logistic regression^a^ Remission (HDRS-24 ≤ 7)		Model 1^b^		Model 2^c^		Model 3^d^	
		OR per 1 SD	p-val	OR per 1 SD	p-val	OR per 1 SD	p-val
***Step***_***1***_	DLPFC(p9-46v)-VIS(MT+)					0.09	0.008
	Intra-VIS(ventral)	0.14	0.01	0.15	0.01	0.17	0.014
***Step***_***2***_	Intra-DMN(10r)	0.10	0.005				
	DLPFC(46)-DMN(s32)			11.56	0.005		
**% Accuracy**		***Step***_***1***_	***Step2***	***Step***_***1***_	***Step2***	***Step***_***1***_	***Step2***
Global^e^		77.8%	100%	77.8%	94.4%	83.3%	94.4%
Non-remitter		77.8%	100%	77.8%	100%	77.8%	100%
Remitter		77.8%	100%	77.8%	88.9%	88.9%	88.9%

^a^Stepwise method. Step_1_: 1-factor; Step_2_: 2-factor. Firth, D. (1993) Bias reduction of maximum likelihood estimates. *Biometrika* **80**, 27–3.

^b^Model 1: Intra-VIS(ventral) and intra-aDMN(10r).

^c^Model 2: Intra-VIS(ventral) and DLPFC(46)-aDMN(s32).

^d^Model 3: DLPFC(p9-46v)-VIS(MT+) and intra-VIS(ventral).

^e^Average accuracy non-remitter and remitter; cut-point 0.5.

DLPFC(p9-46v)-VIS(MT+) connectivity alone predicted 83.3% (88.9% of remitters and 77.8% of non-remitters). The addition of ventral VIS connectivity in Model 3 (Table 3), also increased predictive value to 94% accuracy of remission. In ROC analyses, Models 1–3 (Fig. 5B) accounted for AUC values of 100%. In LOOCV analyses, Models 1–3 showed 0.83–0.89 accuracy (Table 4).

**Table 4:** Leave-one-out cross validation (LOOCV) analyses on significant FDR corrected Models.

Connectivity at baseline			LOOCV analyses		
			Accuracy	95% CI bootstrapping (1000 iteration)	
1-Factor Models	Constant	Intra-VIS(ventral)	0.78	0.56	1
	**Model 1**	**Intra-aDMN(10r)**	**0.89**	**0.61**	**1**
	**Model 2**	**DLPFC(46)-aDMN(s32)**	**0.89**	**0.61**	**1**
	Model 3^b^	DLPFC(p9-46v)-VIS(MT+)	0.72	0.56	1
		DLPFC(p9-46v)-VIS(FST)	0.78	0.56	1
2-Factor Models^a^	Model 1	Intra-VIS(ventral)	0.83	0.83	1
		Intra-aDMN(10r)			
	Model 2	Intra-VIS(ventral)	0.83	0.83	1
		DLPFC(46)-aDMN(s32)			
	**Model 3** ^**b**^	DLPFC(p9-46v)-VIS(MT+)	0.83	0.83	1
		Intra-VIS(ventral)			
		**DLPFC(p9-46v)-MT+ (FST)**	**0.89**	**0.72**	**1**
		**Intra-VIS(ventral)**			

Best Models are highlighted.

^a^All 2-factor Models include intra-VIS(ventral) connectivity.

^b^Model 3 includes connectivity between DLPFC(p9-46v) and the entire MT+ region vs. its most significant parcel (FST).

### Parcelwise analysis

Given the overall significant involvement of reduced connectivity with the MT+ complex and within the ventral VIS region in prediction of ECT response, follow-up analyses explored connectivity related to individual parcels of these regions.

At baseline, within the MT+ VIS region, strongest (negative) correlations were observed between the Fundal area of the Superior Temporal (FST) sulcus (r = −0.66, p = 0.003) or the Lateral Occipital area 1 (LO1) (r = −0.61, p = 0.008) and the DLPFC(p9-46v) **(**Table 1). DLPFC(p9-46v)-FST connectivity was highly predictive of remission (AUC = 0.91, p < 0.001, 95% CI: 0.75–1). By contrast, DLPFC(p9-46v)-LO1 connectivity had only a modest predictive value (AUC = 0.77, p < 0.03; 95% CI: 0.51–0.99).

Within the ventral VIS region, strongest (negative) correlations were observed between the Fusiform Face complex (FFC) (r = −0.52, p = 0.03), area V8 (r = −0.53, p = 0.02) or the PIT complex (r = −0.56, p = 0.015) and the ventral region as a whole (Table 1). The AUC for prediction of remission was highly significant for PIT (AUC = 0.90, p < 0.001, 95%CI: 0.72–1). The predictive value for other parcels within the ventral region - e.g., FFC (AUC = 0.79, p = 0.008, 95%CI: 0.56–0.96) or V8 (AUC = 0.82, p = 0.005, 95%CI: 0.58–1) - were also significant but below for that of PIT.

Two factor models involving specific parcels within MT+ or ventral VIS regions were also assessed with multiple linear and logistic regression. These models were stronger than models involving the overall regions from which the parcels were taken (Supplementary Results).

### Connectivity measures, motion displacement regressors and multiple comparison correction at baseline

No significant correlation between the mean relative motion displacement from frame-to-frame and percentage change in depression scores (r = −0.15, p = 0.55), nor between the mean absolute motion displacement from the first fMRI frame and percentage change in depression scores (r = 0.04, p = 0.87), were observed.

Further inspection of the association between baseline functional connections and change in depression scores revealed no significant effects of motion displacement (mean absolute or mean relative) on correlation values (Table 1). After FDR controlled multiple comparison correction, connectivity within aDMN(10r), or between DLPFC(46) and aDMN(s32), or DLPFC(p9-46v) and VIS(MT+), remained significant (Table 1). Of note, DLPFC(p9-46v)-VIS(MT+) survived multiple comparison correction after adjusting by motion displacement regressors, in line with the notion of distance-dependence bias^58^. Within the MT+ region, connectivity between FST and DLPFC(p9-46v), adjusted by motion displacement regressors, also remained significant after FDR correction (Table 1).

Finally, LOOCV analyses on FDR corrected models showed 0.83–0.89 accuracy (Table 4).

### Changes in connectivity associated with clinical improvement

In order to identify correlates of improvement, changes in depression scores were correlated with change in RSFC measures (Supplementary Table 4). Consistent with predictor models, significant negative correlations were observed between change in DLPFC(46)-sgACC(25) connectivity and change in symptoms (r = −0.63, p = 0.005), suggesting that increases in anticorrelation between the parcels correlate with improvements in symptoms.

**Figure 6:**
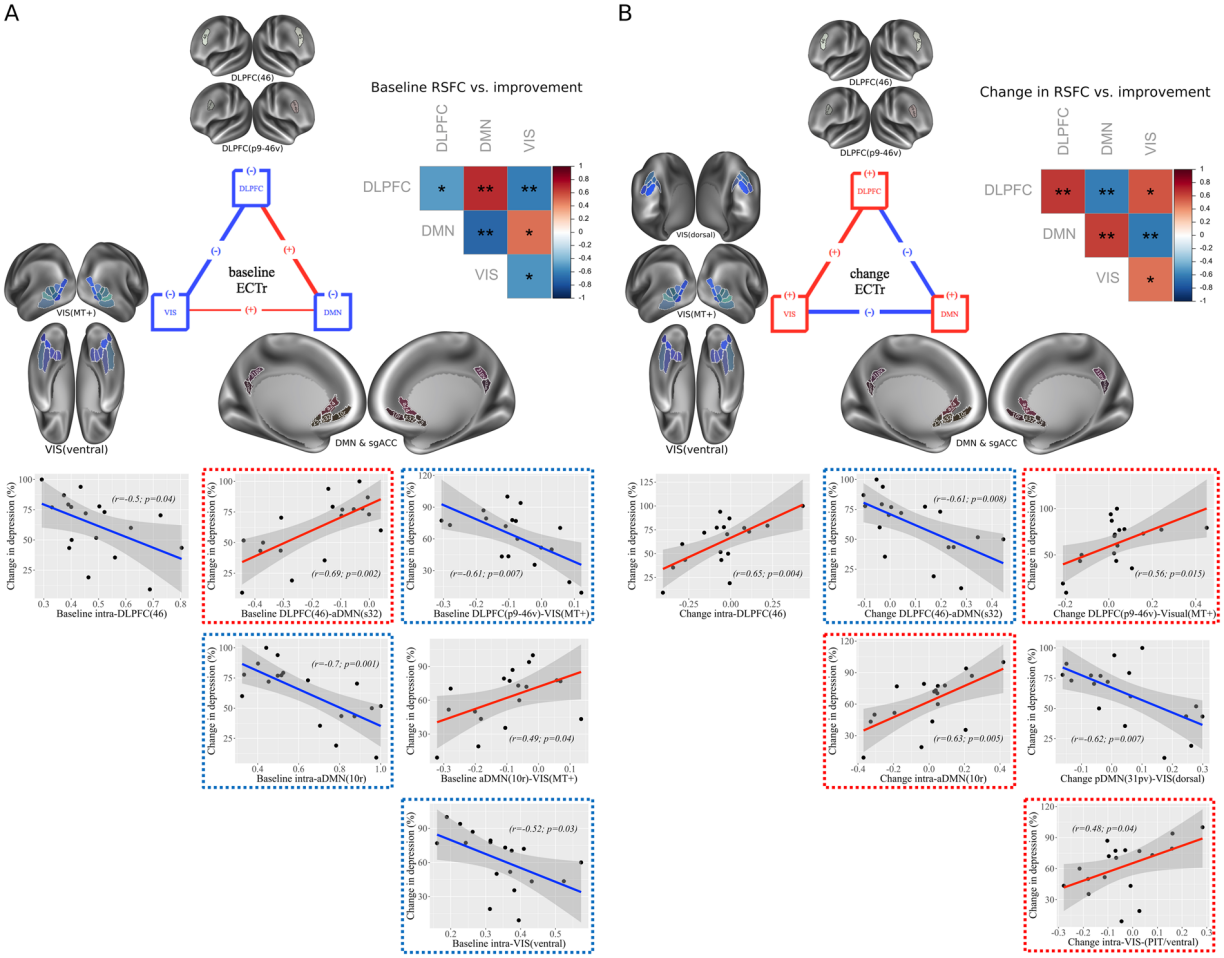
Baseline vs. change within and between DLPFC, DMN and VIS connectivity. (**A**,**B**) (from up-down, left-right) Schematic representation of RSFC structure at baseline (**A**) or change after ECT (**B**) associated with improvement in depression (% change in depression scores) ([pre-post/pre] × 100); colored straight lines show significant connections, with positive (+) or negative (−) RSFC correlation with ECT response. Correlation matrix of pairwise correlation between most significant connections at baseline (A), or change after ECT (**B**), within and between DLPFC, DMN and VIS networks and improvement in depression scores (% change in depression scores) ([pre-post/pre] × 100); colors (red, blue) represent strength of correlations (+1, −1); *p < 0.05, **p < 0.01. Correlation plots with baseline (**A**), or change after ECT (B), RSFC data for within and between DLPFC, DMN and VIS associated with improvement in depression (% change in depression scores) ([pre-post/pre] × 100); colored dot lines show significant connections with positive (+) or negative (−) correlation from final models.

Similarly, change in connectivity between DLPFC(46) and aDMN(a24) (r = −0.63, p = 0.005), aDMN(s32) (r = −0.61, p = 0.008; Fig. 6B) or aDMN(10r) (r = −0.60, p = 0.009) all correlated with response, with increases in anticorrelation between parcels correlating with greater improvement. Similar magnitude correlations were also observed between treatment response and increase in DLPFC(p9-46v) anticorrelation to these same regions (aDMN(a24): r = −0.58, p = 0.01; aDMN(s32): r = −0.52, p = 0.03; aDMN(10r): r = −0.55, p = 0.02).

Consistent with predictor models (Fig. 6A), positive correlations were also observed between treatment response and both increased correlation between DLPFC(p9-46v)-VIS(MT+) (r = 0.56, p = 0.015; Fig. 6B), and increased anticorrelation between VIS(dorsal)-pDMN(31pv) (r = −0.62, p = 0.007; Fig. 6B).

For within-network correlations, significant positive correlations with treatment response were observed for intra-DLPFC(46) connectivity (r = 0.65, p = 0.004; Fig. 6B), or connectivity between DLPFC(46) and DLPFC(p9-46v) (r = 0.69, p = 0.001); for intra-aDMN(10r) connectivity (r = 0.63, p = 0.005; Fig. 6B) or intra-pDMN(31pv/v23ab) connectivity (r = 0.61, p = 0.007); and for intra-VIS ventral connectivity (i.e., between PIT and the ventral region as a whole) (r = 0.48, p = 0.04; Fig. 6B).

### Parcelwise analysis after ECT

We also examined correlation within individual parcels of MT+ and ventral or dorsal VIS regions (Supplementary Results). Within the MT+ region, strongest (positive) correlations were observed between LO1 or FST and DLPFC(p9-46v) (Supplementary Table 4). Within the dorsal stream, strongest (negative) correlations were observed between V3A, V3B, V6A and pDMN(31pv) (Supplementary Table 4).

### Change in connectivity measures, motion displacement regressors and multiple comparison correction

Finally, no significant effects of motion displacement (mean relative or mean absolute) on the association between change in connectivity and depression scores were observed (Supplementary Table 4). After FDR controlled multiple comparison correction, connectivity between DLPFC(46, p9-46v) and aDMN(a24); or between DLPFC(46) and sgACC(25), aDMN(10r), or aDMN(s32); or between VIS(dorsal) and pDMN(31pv); or within aDMN(10r), pDMN(31pv/v23ab) or DLPFC(46, p9-46v/46), all remained significant (Supplementary Table 4).

### Cortical Thickness and RSFC

No significant effect of treatment response on cortical thickness was found. Cortical thickness was not associated with remission nor with treatment response.

## Discussion

This study evaluated RSFC patterns prior to and following ECT, with the goals of both identifying individuals who would benefit maximally from ECT treatment and evaluating RSFC changes associated with successful ECT. As in prior studies of this type^23,59–61^, pretreatment reduced connectivity (decreased negative correlation) within the fronto-limbic network, i.e., between DLPFC(46) and area 25, correlated with the magnitude of subsequent response and significantly predicted which subjects would enter remission. Similar relationships were observed for connectivity between DLPFC and DMN, also consistent with prior observations^59,62–66^; our findings regarding the fronto-limbic and DMN networks are further discussed in Supplementary Discussion.

A novel finding of the present study is that baseline reductions in RSFC between DLPFC and VIS regions, as well as reduced local connectivity within VIS, also significantly predicted subsequent treatment response, and changed along with improvement in depression. Moreover, inclusion of pretreatment connectivity reductions within the ventral VIS region along with other measures in the two-factor prediction algorithm significantly improved the accuracy of prediction and permitted ~100% discrimination between remitters and non-remitters within this sample, and >80% accuracy in a confirmatory LOOCV analysis.

VIS disturbances in TRD have previously been demonstrated using task-based activation and functional connectivity^45,56,67,68^, or metabolite concentration approaches^69,70^, but RSFC with or within VIS regions and treatment response in TRD has not been previously investigated. These findings thus provide strong evidence both for VIS cortical involvement in the pathophysiology of TRD and for the utility of RSFC as a predictor of ECT response in depression.

**Figure 7:**
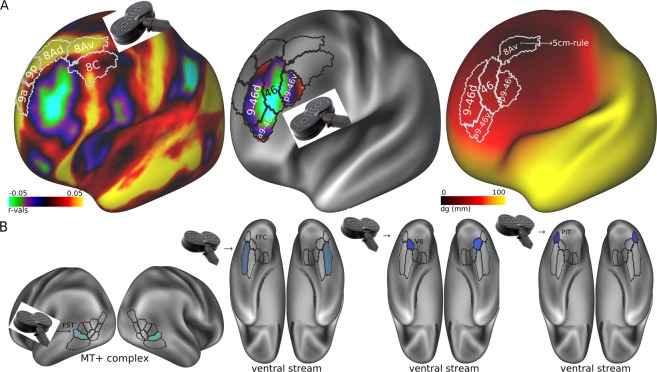
DLPFC_pos_ *versus* DLPFC_neg_ and potential targets for TMS. (**A**) (from left-right) sgACC RSFC map from an independent cohort of 1200 healthy subjects from HCP was generated to identify more correlated (DLPFC_pos_) and anticorrelated (DLPFC_neg_) left DLPFC coordinates in the same sample of healthy subjects; colors represent surface vertices with negative (blue-violet) or positive (red-yellow) correlation with the sgACC. Geodesic map showing the geodesic distance (dg) in mm from average 5-cm MNI coordinates to the rest of the brain. (**B**) Potential VIS targets for FMRI-guided TMS.

In the present study, we used a recently published multimodal parcellation map that permits sub-fractionation of regional networks into constituent components (“parcels”)^2^. This allowed us to identify discrete sub-regions of cortex (e.g., p9-46v, 46, FST, FFC, V8 or PIT; Fig. 7A,B) that may serve as appropriate targets for lower energy, non-invasive^15,71,72^ brain stimulation alternative to ECT; e.g., TMS using figure-8 field coils^73^ is focal (~1.3 cm) and superficial (0.9–3.4 cm). fMRI-guided TMS may allow identification and targeting of circuit-based targets using RSFC between specific DLPFC, DMN or VIS parcels, such as connectivity between DLPFC(p9-46v) and MT+ (FST), or between DLPFC(46) and DMN(s32).

To our knowledge, this is the first study to evaluate RSFC involving VIS cortex as a predictor of treatment response. Although sensory functions were once considered to be intact in neuropsychiatric disorders, over recent years there has been increased documentation of VIS cortical dysfunction in conditions such as schizophrenia^74^, autism spectrum disorder^75^ and aging^76^. Higher order VIS^2^ areas are divided into 3 regions, i.e., dorsal, ventral and MT+ complex. Consistent with our *a priori* hypothesis, pretreatment reduced connectivity (reduced positive correlation) between DLPFC_neg_ and VIS was highly predictive of ECT treatment response including remission (Fig. 2C). Within DLPFC_neg_, parcel DLPFC(p9-46v) showed the highest correlation, while within MT+ complex, parcels FST and LO1 were most involved. Accuracy of models based on VIS connectivity were similar to models involving DLPFC and DMN.

Furthermore, when RSFC involving ventral VIS regions was included in two-predictor models, significant added predictive value was obtained over and above contributions of DLPFC and DMN (Fig. 5B). Thus, reduced positive RSFC within ventral VIS (Fig. 4C) and aDMN(10r) (Fig. 3C), or between DLPFC(p9-46v) and VIS(MT+) (Fig. 2C), or negative RSFC between DLPFC(46) and aDMN(s32) (Fig. 1C), at baseline significantly predicted ECT response.

Pretreatment DMN-VIS reduced connectivity (reduced negative correlation) also significantly predicted remission, although to a lesser extent than DLPFC-VIS connectivity; i.e., reduced connectivity between aDMN(10r) and MT+ VIS (e.g., parcels FST, LO1). After treatment, increased positive RSFC between VIS and DLPFC, or increase negative between VIS and DMN, were highly correlated to symptom improvement (Fig. 6B).

Finally, reduced positive RSFC within-VIS regions at baseline, especially within the ventral stream (e.g. parcels FFC, V8, PIT), was highly predictive of remission, with similar classifier performance (AUC) to that for connectivity between DLPFC-aDMN(s32), DLPFC-VIS(MT+) or intra-aDMN(10r). Following ECT, increased positive correlation within ventral VIS also correlated with magnitude of treatment response (Fig. 6B).

Although the physiological basis for the correlations between VIS connectivity and ECT response are unknown, regions such as MT+ complex are directly or indirectly coupled to parietal and frontal regions associated with sensory-motor integration^77^. Multisynaptic inputs in non-human primates from the frontal cortex to MT+ complex and ventral stream are derived from ventral area 46^78^, which is possibly involved in carrying functionally diverse top-down signals to each VIS region (i.e., dorsal, ventral and MT+).

The MT+ complex incorporates areas in lateral occipital and posterior temporal cortex, and is extensively activated during both motion and social cognition tasks^2^. Within MT+, the connectivity in non-human primates between area FST (dorsal) and the dorsal processing stream directed to posterior parietal cortex is important in spatial aspects of vision, while FST (ventral) connectivity to the ventral processing stream is related to object vision^79^. Thus, the MT+ parcels FST and LO might function as an intermediary of top-down regulation of dorsal and ventral streams of VIS.

The ventral stream incorporates face-sensitive regions such as FFC^2^ and PIT^80^ and color sensitive regions such as V8^81^. Patients with MDD show increased FFC activity to sad faces and decreased activation to happy faces^82^, and slowed face emotion recognition^83^. Moreover, alterations in color processing are known both scientifically^53,84–87^ and colloquially (“seeing the world through grey tinted glasses”)^88^ in depression. More basic deficits in VIS function, such as alterations in sensory event-related potentials or center-surround inhibition of motion processing^89^ are also reported, consistent with our VIS findings.

A surprising result of our study is that the strongest model for prediction of ECT response involved connectivity within ventral VIS by itself, rather than connectivity between VIS and other brain regions, in combination with connectivity within aDMN. Although the basis for the intraregional dysconnectivity is unknown, both serotonergic and GABAergic theories can be considered. In humans, VIS cortex receives dense serotonergic innervation. Serotonergic fibers arise in the raphe nuclei and project anteriorly to innervate inferior and medial prefrontal cortex along with looping posteriorly to terminate in VIS regions. Treatment of depression with the serotonin 5-HT2A receptor agonist psilocybin improves emotional processing^83^, suggesting potential serotonergic mediation of the effects. Additionally, ECT-induced changes in several 5-HT-receptor subtypes^18,90,91^ in the central nervous system, have also been reported.

Occipital GABA levels are also reduced in MDD^92^, consistent with center-surround inhibition abnormalities^89^. Furthermore, such levels increase following ECT^93^. Both serotonin^94^ and GABA^95–97^, may modulate RSFC in healthy individuals. The present study suggests that disruption of intrinsic connectivity within both VIS and DMN may indicate both need for and successful response to treatments such as ECT that may function in part through modulation of serotonergic and GABAergic activity. Thus, to the extent that serotonergic and GABAergic dysfunction is involved in TRD, disturbances in RSFC between VIS cortex and DLPFC/DMN are expected as part of a larger RSFC disturbance.

The findings of impaired connectivity within ventral VIS also converge with reported alterations in attention to happy vs. sad faces in depression. Connectivity within VIS cortex, including ventral VIS, may be modulated by top-down connections from DLPFC and limbic regions such as amygdala or sgACC^98–101^. Thus, even though connectivity was obtained during the resting state, it is possible that decreased top-down DLPFC or increased top-down limbic modulation of VIS may have contributed to the present results. Future studies evaluating modulation of RSFC as a function of attention to affective stimuli are therefore required.

Alternatively, intrinsic abnormalities of ventral VIS connectivity may lead to intrinsic biases in VIS function that cannot be overcome through usual top-down control mechanisms. VIS connectivity may also be indexed by generation of the posterior alpha rhythm, with reduced connectivity associated to an alpha synchronization deficit^102^. Alpha rhythms in parieto-occipital cortex are controlled by top-down signals^103^ and their disruption might lead to a suboptimal state of neural synchronization. Future studies would thus be needed to evaluate the potential utility of resting posterior alpha for prediction of ECT response.

Based upon the present pattern of results, we propose a dysfunction in the hierarchical organization within and between VIS, DMN, and DLPFC_neg_ subnetworks as a new biomarker of ECT response, which also sheds light on underlying mechanisms. Specifically, we observed that TRD patients who show the best response to ECT show a pretreatment pattern characterized by reduced anti-correlation between DLPFC_neg_-DMN and DMN-VIS, as well as reduced positive correlation between VIS-DLPFC_neg_ or within VIS and DMN (Fig. 6A).

Moreover, effective ECT increases the strength of anti-correlations between DMN and DLPFC or VIS, and of the positive correlation between VIS and DLPFC_neg_ (Fig. 6B), or within VIS and DMN. The resting anticorrelation between DLPFC and DMN is extensively studied and serves as the basis for suppression of DMN during active task conditions. Our results suggest that failures of deactivation may result in part from reduced connectivity both within DMN and between DMN and DLPFC. The VIS-DLPFC correlation is also studied in the context of attentional filtering of distracting information (bottom-up/top-down inhibitory control of the distractor related processing). The VIS-DMN anti-correlation is less studied, but may underlie the ability of VIS stimuli also to suppress DMN activity^104^.

Aberrant DMN connectivity is well established in MDD^29,105,106^, including TRD^32,107^. The present findings suggest that ECT may function primarily to restore the normal^108^ anti-correlation of DMN with both “top down” and “bottom up” networks, in order to suppress ongoing DMN hyperactivity that may be associated with increased “mind wandering” relative to goal-directed activity. Positive connectivity between DLPFC and VIS regions is critical for normal goal-directed VIS activity^109^. ECT-induced changes in connectivity may therefore restore both baseline VIS biases and top-down VIS control. Interestingly, our study implicates both face- (e.g. FFC, PIT) and color- (e.g. V8) parcels within ventral VIS, consistent with popular depictions of the disorder.

The only model to obtain 100% accuracy in both logistic regression and ROC analyses involved connectivity within ventral VIS and aDMN(10r) (Model 1, Table 3), or aDMN(s32/10r) (Model 5, Supplementary Table 3). The rostral MPFC (i.e., 10r) is considered a focal point of neural communication, participating in multiple functional networks and enabling high levels of functional diversity (functional hub)^110^. Disturbances of this hub region and their interconnections are likely to cause severe impairments due to their influential role in global integrative processes.

Our results showed abnormal functioning of this hub region was associated to disturbances in the pattern of dynamic interactions (outgoing projections) with central regions of attentional networks, such as DLPFC and VIS, with decreased connectivity intra-aDMN(10r) associated to decreased connectivity intra-DLPFC or DLPFC-VIS, and DLPFC-DMN or DMN-VIS (connected components) (Fig. 5A), reinforcing the central role for 10r hub node in the overall network structure and dynamical organization. Depression has also been associated with abnormal topological organization of brain networks, including disrupted global integrity and regional connectivity^111^.

On the other hand, decreased intra-VIS connectivity was only associated to decreased intra-DLPFC connectivity (Fig. 5A), consistent with the observation of primary regions (e.g., VIS) to participate in small number of functional networks compared to the participation of 10r in multiple functional networks^110^. Consistent with current results, Zhang *et al*.^112^ showed decreased regional connectivity (degree efficiency and betweenness) in the DLPFC and occipital regions. As such, the model combining both, RSFC within 10r and ventral VIS, includes two core non-overlapping components of the overall network dysfunction in TRD.

The posterior alpha network comprises the occipital lobe and parts of the temporal and medial posterior-parietal cortex (including posterior cingulate gyrus and precuneus) and temporal lobes^113^. After effective treatment, connectivity changes within (increased correlation) and between VIS and pDMN (increased anticorrelation) were associated with good response. Connectivity changes propagated through the entire VIS network including MT+ complex (e.g., FST, L03, V3CD), dorsal stream (e.g., V3A, V3B, V6A, V7) and ventral stream (e.g., PIT), extending to pDMN (e.g., 31pv, v23ab), suggesting modulatory effects in the intrinsic activity of VIS system.

In addition to predicting ECT response, the present results argue for a move in scalp location of the standard TMS probe placement ≥ 20–50 mm of geodesic distance (*dg*) (defined as the length of the shortest directed path from the current canonical location) from the present location which is defined based upon the 5cm-rule (corresponding to vertex 30641) (Fig. 7A). Furthermore, it argues strongly for individual targeting of rTMS to areas 46 or p9-46v, which correspond to the parcels of DLPFC region that are anti-correlated to sgACC (DLPFC_neg_). These findings are consistent with those of Fox *et al*.^15,71,72^, who also suggested that the DLPFC_neg_ (BA46) region may serve as a critical treatment target in depression, and support the role of DLPFC-sgACC, DLPFC-aDMN, and DLPFC-VIS RSFC both as predictors of need for ECT, and correlates of change.

There are several limitations of the present study. First, the sample size is relatively small, necessitating replication in larger cohorts. There were also no cohorts receiving other treatment types (e.g. antidepressants, ketamine) so that it is unknown whether the network effects predict general treatment response or response specifically to ECT.

Second, in order to obtain 100% correct classification, we required use of two independent predictors. Because the model was not pre-specified, the present results must be considered exploratory and must be confirmed in a future sample. Nevertheless, analyses were constrained using a pre-planned parcellation scheme, which limited the number of comparisons. Specifically, DLPFC_neg_, sgACC, aDMN, ventral VIS and MT+ complex were all identified *a priori*. DLPFC_neg_-sgACC and DLPFC_neg_ were selected for analyses based upon prior literature. Although 3 VIS regions were used in analyses, the findings regarding DLPFC-MT+ connectivity were sufficiently strong to survive FDR correction for multiple comparisons. The findings with intra-ventral connectivity were weaker, but were additive to several other connectivities (DLPFC-sgACC, DLPFC-aDMN, DLPFC-VIS, intra-aDMN), providing convergent support. Predictive models showed 0.83–0.89 accuracy in LOOCV analyses even following control by motion regressors and FDR correction, and thus support further studies with this algorithm.

As opposed to the present study, a recent study^114^ evaluated changes in patients with schizophrenia treated with combined antipsychotics and ECT, and showed a five-predictor algorithm (i.e., DMN, temporal lobe network, language network, cortico-striatal network and fronto-parietal network) that correctly classified 83.8% of 13 patients, suggesting that multiple predictors do not, of necessity, produce high predictive values. Here, by uniquely incorporating VIS RSFC measures in our algorithm, we obtain 100% correct classification in a sample of comparable size and with only one- or two-predictors.

Finally, since there is not a multi-modal subcortical parcellation available at this time, we considered only cortical structures in the present study. By contrast, a recent study demonstrated that DLPFC-striatal connectivity significantly predicted TMS response^115^. Thus, future studies incorporating both cortical and subcortical structures are needed.

## Methods

### Participants

We studied 18 TRD patients receiving ECT for clinical indications at New York State Psychiatry Institute (NYSPI) or Columbia University Medical Center (CUMC) (ages: 18–75; mean = 52, SD = 12), who met DSM-IV criteria for a Major Depressive Episode (MDE) according to the diagnostic assessment by the Structured Clinical Interview Patient Edition (SCID-P), with scores of 18 or greater (mean = 26.5, SD = 3.9) on the 24-Hamilton Depression Scale (HDRS-24). Patients with comorbid other Axis I or Axis II psychiatric disorders were excluded. All subjects were right-handed and without severe medical conditions. The NYSPI/CUMC Institutional Review Board approved this study. All participants provided written informed consent. This study was performed in accordance with all relevant guidelines and regulations.

### Electroconvulsive therapy

Participants received a full course of right unilateral, frontal ECT. ECT and anesthesia procedures complied with APA Guidelines^116^. Blood pressure, pulse, ECG, and pulse oximetry were monitored prior to anesthetic induction and continuously during the procedure. Seizure manifestations were recorded with two frontal-mastoid EEG channels, as well as motor manifestations with the cuff technique. Using conservative criteria (≥15 seconds), generalized seizures of adequate duration were elicited at each treatment. Ultra-brief right unilateral (RUL) ECT was given with a Thymatron System IV brief pulse device. Seizure threshold (ST) was determined at the first treatment using the dose titration method^117^. Dose at subsequent treatments was at 6xST or maxim output, whichever was lower. Patients remained on pre-existing antidepressant medication without alteration over the course of the study.

The Hamilton^118^ rating scale (HDRS, 24 items) was obtained pre/post ECT and used for outcome analyses.

### fMRI Data Acquisition and Processing

High resolution anatomical images and resting-state functional MRI were collected pre/post ECT. Post study imaging was performed approximately 48–72 hours after the last treatment to allow sufficient time for recovery from treatment. Anatomical and functional imaging data from each subject were collected and processed using acquisition guidelines and processing pipelines provided by the Human Connectome Project (HCP)^119^. Collected data was also compared to the healthy individuals from the HCP database (https://www.humanconnectome.org/study/hcp-young-adult/data-releases). High-resolution functional imaging scans were completed at the MRI facility at NYSPI using a GE Discovery MR750 3.0 Tesla full body MR system equipped with a 32-channel phased array head coil (Nova Medical, Wilmington MA).

Subjects were placed in the scanner with head cushioning to restrict head movement. Following localizer scans (5 minutes), distortion correction scans (B0 fieldmap), a pair of T1-weighted images, and a T2-weighted image are acquired over 25 minutes, followed by one 10-minute resting state fMRI scan. Total scan time was 45 min. Sessions were conducted prior to and following completion of the course of ECT. T1-weighted images acquired for anatomical co-registration were transverse T1-weighted BRAVO sequence with the following acquisition parameters: 3D sagittal, 0.8 mm isotropic, matrix size = 300 × 300, slices = 220, TR = 7.856 ms, TE = 3.108 ms, flip angle = 12°, TI = 450 ms and CUBE T2-weighted image was acquired with these parameters; 3D sagittal, 0.8 mm isotropic, matrix = 320 × 320, # of slices = 220, TR = 2500 ms, TE = 95.708 ms, flip angle = 90°.

Functional images were acquired with a GE-EPI sequence (2.5 mm isotopic, slice plane = transverse, TR = 2500 ms, SENSE factor = 2, TE = 22 ms, matrix = 96 × 96, slices = 54, phase encode = A −> P); an instruction was given to all patients before the functional MRI sequence to have their eyes opened. Images were transferred to a workstation with the HCP processing pipeline version 3.4 installed. The pipeline implements standard automated structural and fMRI processing (movement correction, atlas realignment, creation of cortical surface model etc.) with FSL 5.0.6, FreeSurfer v5.3.0-HCP, and Connectome Workbench v1.0, and two additional procedures that improve upon standard preprocessing: required use of field-mapping sequences to “undistort” GE-EPI images and creation of a “gray-ordinate” CIFTI-format files that only contained data from cortical and subcortical gray matter. The HCP pipelines were modified to a) process the B0 fieldmaps produced by the GE MR scanner and b) to implement slice-time correction (fMRIVolume processing pipeline set to ODD for interleaved).

Resulting structural and functional data were aligned in volume space to the MNI152 atlas and in surface space to the HCP-generated Conte69 surface atlas based on cortical folding patterns. Resting-state data files have undergone removal by regression of CSF, white matter, whole brain, and movement parameters (6 translation/rotation parameters + quadratic combinations of the 6 parameters + derivatives of these 12 parameters); to avoid slice-time correction affecting the movement parameters, movement parameters were derived from the data before HCP processing^120^. Frames with FD >0.2 mm and/or DVARS with >(75percentile) + (1.5) × (interquartile range) (fsl defaults) were then censored and interpolated^121,122^, and then a bandpass filter with a low cutoff of 0.0005 Hz and high cutoff of 0.2 Hz was applied. Subsequent analyses only used the non-censored frames (they did not include the interpolated frames).

Even though the most common approach for removing global structured noise is to remove the mean (across space) fMRI timecourse from the data using global signal regression^123^, there are some limitations associated to this approach given that global signal may differ between patients with psychiatric disorders^124,125^, and because the removal of neural signal may distort the resulting connectivity measures in network-specific ways^126^.

### Definition of ROIs

Regions of interest (ROI) for these analyses were defined using the multi-modal parcellation^2^ of human cortex (Supplementary Table 5); all ROIs used for analyses were bilateral. DLPFC, DMN and VIS networks were created manually following grouped regions from the multi-modal parcellation (Supplementary Fig. 1A–C).

The sgACC ROI (area 25) was first used as a seed on an independent cohort of 1200 healthy subjects from the HCP database. The sgACC RSFC map generated across 1200 healthy subjects from the HCP dataset was used to find parcels within the DLPFC region^2^ with highest negative (DLPFC_neg_) correlation with the sgACC (Supplementary Fig. 2), based on previous findings^15^. These negative areas (46, p9-46v, a9-46v, 9-46d) within the DLPFC were used to create our DLPFC_neg_ masks.

Regions within the anterior (aDMN: a24, s32, 10r) or posterior (pDMN: 31pv, v23ab) medial locus of DMN were selected based on their strongest association in the resting-state with task negative, as shown in Glasser *et al*.^2^. Main parcels within rostral ACC (a24, s32) were also selected based on early studies on predictors of treatment response^64,127^. MNI coordinates from the core set of hubs, rostral MPFC (−6 52 −2) or PCC (−8 −56 26) based on previous work^36^, were approximately^128^ mapped to Glasser parcels rostral MPFC (10r; surface vertex 28169) or PCC (31pv, v23ab; surface vertex 26238), respectively. These cortical areas were used to create our aDMN or pDMN masks.

The VIS network has been divided into primary VIS (V1), early VIS (V2-V3), dorsal stream, ventral stream and MT+ complex regions. We exclusively focused on higher order VIS regions, grouped into three separate subsystems: dorsal and ventral streams and MT+ complex. These separate subsystems were used to create our VIS masks.

### Seed-based RSFC analyses

Baseline RSFC maps of areas within the DLPFC_neg_ (46, p9-46v, a9-46v, 9-46d) were first created for each TRD patient to explore patterns of connectivity with sgACC (25) and rostral ACC (a24) as predictors of treatment response. Cortical areas within the DLPFC_neg_ with predictive power (46, p9-46v) were then selected to further explore patterns of connectivity with DMN and VIS networks.

RSFC maps of areas within the aDMN (s32, 10r) and pDMN (31pv, v23ab) were created for each TRD patient to further explore patterns of connectivity with VIS network.

RSFC maps of each separate region of VIS network (dorsal, ventral and MT+) were created for each TRD patient to explore patterns of intra-VIS connectivity (intra-regional connectivity). First, the spatial map of each VIS region was used as the predefined mask. Then, RSFC of each VIS region to all voxels in each predefined mask was computed and averaged as the intra-regional connectivity (intra-ventral, intra-dorsal and intra-MT+). Following detection of significant within regional correlations, secondary analyses evaluated the relative contribution of individual parcels within each region by computing parcel-to-region connectivity within region (e.g., FFC to ventral).

### Network analyses

Initial RSFC analyses explored patterns of connectivity between 4 cortical areas within the DLPFC_neg_ (46, p9-46v, a9-46v, 9-46d) and 2 cortical areas within the sgACC (25) or the rostral ACC (a24); 8 correlation analyses were conducted. Subsequent RSFC analyses were based on a network configuration of 9 ROIs: 2 cortical areas within the DLPFC_neg_ (46, p9-46v), 2 cortical regions within aDMN (s32, 10r), 2 cortical regions within pDMN (31pv, v23ab), and 3 VIS subsystems focused on higher order VIS regions (dorsal, ventral and MT+). Using the RSFC measures obtained from this network, we analyzed (1) within-network RSFC, averaged across hemispheres, and (2) between-network RSFC, averaged across hemispheres; 38 correlation analyses were conducted. Analyses focused first on prediction of the degree of treatment response and remission across individuals; and subsequently on correlates of change. Follow-up VIS analyses focused on significant patterns of connectivity between DLPFC(p9-46v) or DMN(31pv) and VIS(MT+, dorsal), or within VIS(ventral), to evaluate the relative contribution of individual parcels within each VIS region (7 parcels for ventral, 6 parcels for dorsal, 9 parcels for MT+).

Fisher’s z transform was applied to individual RSFC maps before group level analyses; uncorrected p-values were reported as only 8 (for initial analyses) and 38 (for network-analyses) comparisons were made; corrected p-values using FDR^129^ controlled multiple comparison correction were computed to see whether any of the uncorrected p-values survived. Leave-one-out cross validation (LOOCV) analysis was conducted on FDR corrected predictive models to estimate potential predictive value when applied to larger samples.

### Influence of motion regressors on depression scores and connectivity

We evaluated if depression scores or connectivity measures were linked to motion estimators. The HCP pipeline provides an estimate of average displacement from initial frame (mean absolute) and frame-to-frame (mean relative) displacement for each run and fMRI session. We evaluated linear trends between percentage change in depression scores and subject’s motion displacement values (Corr), or between percentage change in depression scores and baseline/change in connectivity measures controlling by subject’s motion displacement values (PCorr). Corrected p-values using FDR^129^ were also computed to see whether any of the uncorrected p-values, adjusted by subject’s motion displacement values, survived.

### Statistics

All analyses were conducted using R-package (version 3.5.1). To investigate association between continuous variables, Pearson’s correlation and partial correlation analyses were used. Partial correlation analyses were adjusted by motion displacement values (mean absolute and mean relative).

Initial analyses were performed using simple correlations between each pretreatment RSFC measure and percent change in depressive symptoms, defined as percentage change in HDRS-24 ([pre-post]/pre] × 100%). Multiple linear regression analyses were then conducted to evaluate the potential additive value of significant functional connections to identify best two-factor predictive models of percentage change in HDRS-24. The ability of pretreatment RSFC patterns to predict symptom remission, defined as final HDRS-24 score ≤ 7, was assessed using multiple logistic regression and the area under the receiver operating characteristic (ROC) curve. ROC analyses were conducted using R-package pROC (version 1.8)^130,131^; confidence interval (95% CI) used bootstrapping (2000 stratified bootstrap replicates). Leave-one-out cross validation (LOOCV) analysis was conducted on FDR corrected predictive models; confidence interval (95% CI) of accuracy measure used bootstrapping (1000 iteration). Subsequent analyses used simple correlation analyses to asses ECT-induced changes on baseline connectivity measures by computing correlation between (post-pre) connectivity measures and percent change in depressive symptoms. For two group comparisons, two-sample t-test were used. Sample descriptive statistics for continuous variables are reported as mean ± s.e. All statistics are two-tailed, with preset alpha level for significance of p < 0.05, and n value of 18.

### Cortical Thickness and RSFC

To confirm that changes in cortical thickness did not affect RSFC analyses, cortical thickness was evaluated (Supplementary Methods) as a function of both time (pre/post ECT) and group (remitter/non-remitter), using repeated ANOVA. In addition, we computed correlation analyses between changes in cortical thickness and improvement in depression. Across analyses, no significant relationships were observed between cortical thickness and ECT response.

## Supplementary information

Supplementary Documentation for: Resting state functional connectivity predictors of treatment response to electroconvulsive therapy in depression.
