# Supplementary material for: Resting state functional connectivity predictors of treatment response to electroconvulsive therapy in depression
Source: Sci Rep. 2019 Mar 25;9:5071. doi: 10.1038/s41598-019-41175-4 (PMC6433903; doi:10.1038/s41598-019-41175-4)
Supplement: Supplementary file 1 — Supplementary Documentation for: Resting state functional connectivity predictors of treatment response to electroconvulsive therapy in depression. [file 41598_2019_41175_MOESM1_ESM.docx]

## **Supplementary Documentation for: Resting state functional connectivity predictors of treatment response to electroconvulsive therapy in depression.**

Moreno-Ortega M*^1,2^, Prudic J^1^, Rowny S^1^, Patel GH^1^, Kangarlu A^4^, Lee S^3^, Grinband J^1^, Palomo T^2,5^, Perera T^1^, Glasser MF^6^ and Javitt DC^1^.

Supplementary Results

#### DLPFC-sgACC/rostral ACC:

The degree of anticorrelation between DLPFC(46) and sgACC(25) was related to subsequent treatment response, such that reduced anticorrelation at baseline was associated with greater clinical improvement (r=0.54, p=0.02) and significantly predicted remission (AUC=0.85, p=0.006, 95% CI: 0.65-1). For other DLPFC parcels (i.e., p9-46v, a9-46v, 9-46d), connectivity with sgACC did not predict treatment response.

Baseline DLPFC(46)-aDMN(a24) connectivity (reduced anticorrelation) also correlated with treatment response (r=0.58, p=0.01). While significant, the ability to predict remission remained relatively low (AUC=0.74, p=0.03, 95% CI: 0.47-0.96). A significant correlation (r=0.52, p=0.03) was also observed between DLPFC(p9-46v)-aDMN(a24) connectivity and treatment response. The predictive value of the DLPFC(p9-46v)-aDMN(a24) connectivity (AUC=0.88, p=0.008, 95% CI: 0.68-1) was similar to that for DLPFC(46)-sgACC(25). For other DLPFC parcels (i.e., a9-46v, 9-46d), connectivity with aDMN(a24) did not predict treatment response.

### DMN-VIS:

For DMN-VIS connectivity, a significant positive correlation with treatment response was found for aDMN(10r)-VIS(MT+) (r=0.49, p=0.04**).** AUC for aDMN(10r)-VIS(MT+) (AUC=0.83, p=0.01, 95% CI: 0.59-1) significantly predicted remission. For other aDMN(s32) or pDMN(31pv, v23ab) areas, connectivity with VIS did not significantly predict treatment response.

#### Within-network connectivity:

For DLPFC, reduced connectivity within area 46 (r=-0.50, p=0.035) and between areas p9-46v and 46 (r=-0.49, p=0.039) correlated with treatment response. Both within- (AUC=0.84, p=0.005, 95% CI: 0.64-0.99) and between- (AUC=0.85, p=0.007, 95% CI: 0.62-1) intra-DLPFC connectivity predicted remission.

Reduced connectivity between aDMN(s32) and aDMN(10r) (r=-0.55, p=0.02), correlated with clinical improvement. The predictive value between aDMN(s32) and aDMN(10r) (AUC=0.80, p=0.003, 95% CI: 0.55-1) was also significant but remained below of that for within-aDMN(10r).

Correlations within the dorsal stream (r=-0.46, p=0.055) or MT+ complex (r=-0.36, p=0.14) were not significant; despite the lack of overall correlation with improvement, the AUC for prediction of remission was significant for connectivity within both dorsal stream (AUC=0.86, p=0.003, 95% CI: 0.67-1) and MT+ complex (AUC=0.80, p=0.007, 95% CI: 0.56-0.98).

#### Two Factor Models for prediction of magnitude of improvement:

Significant models were also observed involving other DLPFC, sgACC and DMN, but were somewhat weaker. Specifically, the combination of intrinsic ventral VIS connectivity with either DLPFC(46)-aDMN(a24) (**Supplementary Table 2**, Model 4) or intra-aDMN(s32/10r) (**Supplementary Table 2**, Model 5), accounted for ~55% of the variance, while intrinsic ventral VIS connectivity and DLPFC(p9-46v)-DMN(a24) (**Supplementary Table 2**, Model 6), or DLPFC(46)-sgACC(25) (**Supplementary Table 2**, Model 7), accounted for ~50%.

#### Two factor models for prediction of remission:

The combination of intrinsic ventral VIS connectivity and aDMN(10r/s32) connectivity in Model 5 (**Supplementary Table 3**), increased predictive value to 100% accuracy for both remitters and non-remitters. In Models 4 and 6 (**Supplementary Table 3**), the addition of either DLPFC(46)-DMN(a24) or DLPFC(p9-46v)-DMN(a24) also improved predictive value, with 94% accuracy of remission.

Finally, the model combining DLPFC(46)-sgACC(25) in addition to intrinsic ventral VIS connectivity (Model 7, **Supplementary Table 3**), also improved predictive value but remained below of that for Models 4-6, with 88.9% accuracy of remission.

### Parcelwise analysis:

Within the ventral stream, other areas that showed reduced connectivity with the rest of the ventral visual areas, and significantly predicted treatment response were the Ventro-Medial Visual areas 1 (VMV1) (r=-0.53, p=0.02) or 3 (VMV3) (r=-0.49, p=0.04). The predictive value for other within ventral areas were also significant but below for that of PIT; e.g., between VMV1 (AUC=0.82, p=0.01, 95% CI: 0.59-0.98) or VMV3 (AUC=0.83, p=0.003, 95% CI: 0.60-1) and the ventral stream.

#### Two Factor Models for prediction of magnitude of improvement:

For two factor models for prediction of magnitude of improvement related to ventral VIS, greatest involvement was observed for FFC, V8, VMV3 and VMV1 connectivity. For example, reduced VMV3/ventral connectivity (partial r=-0.55, p=0.022) and intra-DMN(10r) (partial r=-0.73, p=0.001) significantly predicted 64% of the variance in treatment response.

Similarly, VMV3/ventral (partial r=-0.59, p=0.012) and DLPFC(46)-DMN(s32) (partial r=0.74, p=0.001) significantly predicted 66% of response variance. Analogous models with V8/ventral (partial r=-0.53, p=0.03) and intra-DMN(10r) (partial r=-0.70, p=0.002), or V8/ventral (partial r=-0.54, p=0.024) and DLPFC(46)-DMN(s32) (partial r=0.69, p=0.002) also showed significant additivity and predicted 63% of the variance in treatment response.

For two factor models for prediction of magnitude of improvement related to MT+ complex, only LO1 and area FST showed significant independent involvement in response prediction. Specifically, a model including connectivity between DLPFC(p9-46v) and LO1 (partial r=-0.70, p=0.002) along with V8/ventral connectivity (partial r=-0.65, p=0.005) accounted for 63% of the variance, while a model including DLPFC(p9-46v)-FST (partial r=-0.69, p=0.002) and VMV1/ventral connectivity (partial r=-0.58, p=0.014), also accounted for 63% of the variance.

#### Two factor models for prediction of remission:

In logistic regression analyses, within VIS ventral (VMV3, V8) connectivity alone predicted 77.8% (66.7% of remitters and 88.9% non-remitters). Other within VIS ventral (PIT) connectivity, or between DLPFC(p9-46v)-VIS(FST), alone predicted 83.3% (77.8% of remitters and 88.9% non-remitters).

The addition of intra-DMN(10r) connectivity to VMV3/ventral or V8/ventral connectivity increased predictive value, with 94% accuracy of remission. The addition of DLPFC(46)-DMN(s32) connectivity to VMV3/ventral connectivity also increased predictive, with 100% accuracy of remission, while the addition of DLPFC(46)-DMN(s32) to V8/ventral showed 94% accuracy.

For MT+, the combination of DLPFC(p9-46v)-FST connectivity along with FFC/ventral or VMV1/ventral also improved predictive value to 94% accuracy of remission.

### Changes in connectivity associated with clinical improvement:

For DLPFC(p9-46v), but not DLPFC(46), additional correlations were observed for change in connectivity to pDMN(31pv) (r=-0.57, p=0.01) and treatment response. Also, correlations were observed between pDMN(31pv) and VIS(MT+) (r=-0.53, p=0.02).

For within-network correlations, significant positive correlations with treatment response were also observed for connectivity between aDMN(s32) and aDMN(10r) (r=0.59, p=0.01).

### Parcelwise analysis after ECT:

We also examined correlation between DLPFC(p9-46v) and individual parcels within MT+. The greatest correlation was observed for LO1 connectivity (r=0.65, p=0.004). Connectivity between DLPFC(p9-46v) and FST (partial r=0.57, p=0.015) also correlated significantly with response.

Correlations involving pDMN(31pv) and parcels within both MT+ and dorsal VIS were also observed. For MT+, significant negative correlations with treatment response were observed for areas FST (r=-0.49, p=0.04), LO3 (r=-0.54, p=0.02) and V3CD (r=-0.54, p=0.02). For dorsal VIS, ECT-induced reductions in connectivity between pDMN(31pv) and areas V3B (r=-0.61, p=0.007), V3A (r=-0.59, p=0.01), V6A (r=-0.55, p=0.02) or V7 (r=-0.49, p=0.04) all correlated with better treatment response.

Supplementary Discussion

The involvement of sgACC (area 25) in the pathophysiology of depression and as a predictor of response has been extensively studied. For example, in prior studies both sgACC volume^1^ and BOLD fluctuations^2^ significantly predicted ECT response, sgACC activity is also reduced after effective antidepressant treatment^3^. It is postulated that DLPFC hypometabolism might be secondary to limbic hyperactivity, and that DLPFC connectivity (negative correlation) with limbic regions are responsible for antidepressant response^4^.

Evidence for an early antidepressant response at the limbic level followed by a secondary treatment effect in the PFC has been reported^2,5,6^, suggesting an interaction between intra-limbic and limbic-PFC connectivity. Modulation of RSFC patterns of DLPFC are crucial in order to achieve therapeutic response with ECT^7-9^. In this context, a reduction of intra-limbic hyperconnectivity (sgACC), which may then allow the DLPFC to regain control of limbic system activity (and other brain networks) has been suggested^10^. The sgACC is a node within the limbic network. Present results are therefore consistent with theories that alterations in DLPFC connectivity with limbic regions are responsible for antidepressant response^6,11,12^.

sgACC maintains monosynaptic connections with specific prefrontal (orbital, lateral and medial; including DLPFC)^12-14^. In healthy individuals^4^, sgACC shows significant negative connectivity with different regions of DLPFC focused around Brodmann areas 9 and 46. We observed anti-correlated regions of DLPFC involving areas 46, p9-46v, a9-46v and 9-46d, which we term DLPFC_neg_. We also observed correlated regions involving areas 8C, 8Av, 8Ad, 9p, 9a, of DLPFC (DLPFC_pos_), corresponding to Brodmann area 8. MNI coordinates (-41, 16, 54; surface vertex 30641) of the average 5-cm^4^ based targeting approach (FDA TMS protocol) approximately mapped to area 8Av from the multimodal parcellation^15^ within DLPFC_pos_, rather than DLPFC_neg_. The degree to which such stimulation also engages DLPFC_neg_ might be variable across individuals.

We found correlations between treatment response and baseline connectivity (decreased negative correlation) between DLPFC_neg_ (areas 46 and p9-46v) and areas of the aDMN, especially a24 and s32 within rostral ACC. Of these, connectivity between DLPFC(46) and aDMN(s32) showed the highest predictive value. Pretreatment connectivity (decreased positive correlation) within-aDMN(10r), or between aDMN(s32) and aDMN(10r); or within DLPFC_neg_ (e.g., 46), or between DLPFC_neg_ areas (e.g., p9-46v and 46), also significantly predicts treatment response.

Numerous studies have investigated RSFC in relationship to depression. In a systematic review of resting-state functional MRI studies in MDD, an abnormal functional connectivity between DLPFC and DMN was found to contribute to the pathophysiology of MDD^16^. The DMN has also been found to be impaired in first-episode, drug-naïve MDD patients^17-21^. Within aDMN(10r, s32/10r) or between aDMN (a24, s32) and DLPFC connectivity are also consistent with previous findings that showed activity of rostral MPFC (e.g., 10r) and rostral ACC (e.g., a24, s32) had the strongest predictive value for treatment response^72-76^. Moreover, an early study on predictors of treatment response suggested that increased rostral ACC (BA 24a/b) activity rather than sgACC activity predicted response to antidepressant treatment^22^. As opposed to sgACC, rostral ACC is considered a component of aDMN.

To our knowledge, only four prior studies have evaluated changes in RSFC pre/post successful ECT. Consistent with the present results, one study^9^ of 9 individuals reported a negative correlation between a cluster of voxels within Brodmann areas 44, 45, and 46 and medial cortex structures within the aDMN (BAs 24 and 32) following ECT. The coordinates of this cluster (MNI: -38, 32, 34) approximately mapped to Glasser areas 46 and p9-46v.

A second study^7^ of 12 individuals observed changes in RSFC between DLPFC or dorsal MPFC and medial/posterior regions of DMN (pDMN), but not sgACC. However, RSFC between pDMN and DLPFC increased from negative to a positive correlation during the ECT series. Differences between the second study and the present results may be explained by: (1) their older sample and age-related decreased anticorrelation between these networks; (2) ICA vs. seed-based approach, seed-based RSFC measures are the sum of ICA-derived within network and between network connectivities^23^.

A third study^24^, revealed two resting-state networks with significant classification accuracy. A network centered in the dorsal MPFC but also including DLPFC, orbitofrontal and posterior cingulate cortices showed a sensitivity of 84% and specificity of 85%. Another network centered in ACC but also including DLPFC, sensorimotor cortex, parahippocampal gyrus and midbrain showed a sensitivity of 80% and a specificity of 75%. These values are similar to those obtained using DMN-based measures in the present study, supporting DMN involvement in both the pathophysiology of TRD and ECT treatment response.

A fourth study^25^, showed decreased network coherence in a large area of the pDMN (precuneus and angular gyrus) to the network as a whole compared with healthy controls. After ECT, the network coherence was normalized in responders but persisted in non-responders. In our present results, changes in connectivity after effective ECT were found within both aDMN and pDMN (i.e., between areas 31pv and v23ab), with increased within network connectivity associated with better response.

The DMN includes regions that deactivate during processing of external stimuli^26,27^ and activate by introspective cognitive processes^28^. Reduced DMN deactivation during executive tasks is associated with depressive symptoms such as difficulty with inhibition of rumination, concentration and decision-making^29,30^. Furthermore, higher activation within the DMN during executive tasks in depressed subjects might reflect a failure of deactivation^31-33^.

Following ECT, increased anticorrelation between DLPFC(46, p9-46v) and aDMN(a24, s32, 10r), extending to pDMN(31pv), correlated with the magnitude of treatment response, suggesting widespread modulation of DLPFC_neg_-DMN connectivity. Also, increased correlation within aDMN(10r, s32/10r), pDMN(31pv/v23ab/) or DLPFC_neg_(46, p9-46v/46), were highly correlated to treatment response. Disengagement from internally focused thought processes rely on interactions between DMN and DLPFC. The DLPFC is associated with external attention and task execution^34^. Based on these findings it is reasonable to speculate that the increased anticorrelation between DLPFC and DMN found in our study reflects an increased ability for mutual inhibition between DLPFC and DMN networks.

Supplementary Methods

### *Cortical Thickness and RSFC:*

Cortical thickness maps generated with the HCP pipeline were used to extract the mean average of total cortical surface and DLPFC_neg_ (46, p9-46v, a9-46v, 9-46d) region at baseline and after ECT.

|  |
| --- |
| **Suppl. Fig. 1. The HCP’s multi-modal parcellation v1.0 used in RSFC analyses.** (A) Areas of DLPFC_neg_. (B) Areas of anterior/posterior medial locus of DMN and sgACC. (C) Higher order VIS regions. |

| 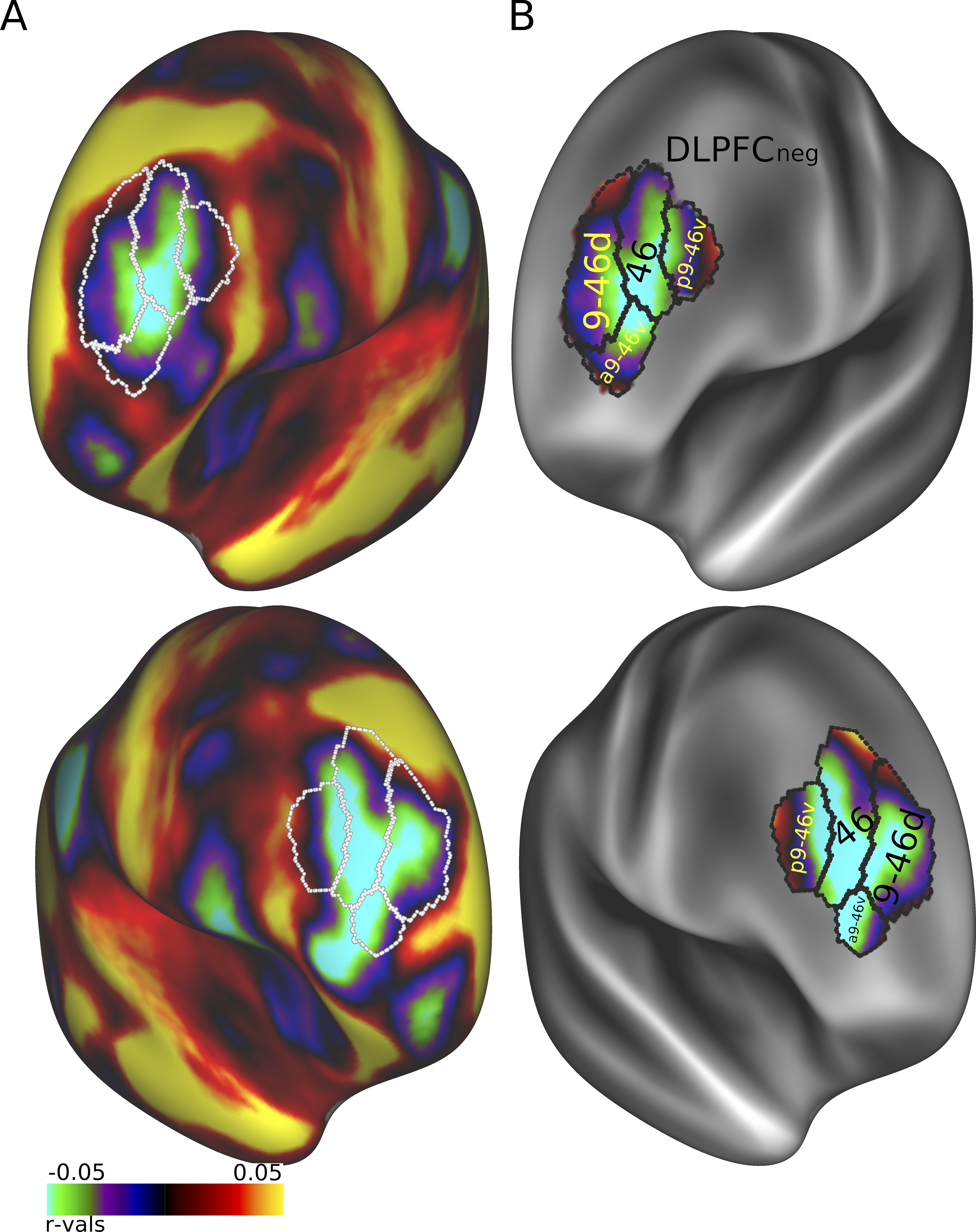 |
| --- |
| **Suppl. Fig. 2. DLPFC_neg_ nodes.** (A) The sgACC ROI from the HCP’s multi-modal parcellation v1.0 (area 25) was used as a seed on an independent cohort of 1200 healthy subjects from the Human Connectome Pipeline. (B) DLPFC nodes were selected based on their anticorrelation with sgACC (DLPFC_neg_). Colors represent surface vertices with negative (blue-violet) or positive (red-yellow) correlation with the sgACC. |

| **Linear regression** | | **Model DLPFC-sgACC** ^a^ | | | | **Model DLPFC-rostral ACC** ^a^ | | | | **Combined** ^b^ | | | |
| --- | --- | --- | --- | --- | --- | --- | --- | --- | --- | --- | --- | --- | --- |
|  |  | *Coefficients* | | *Change statistics* | | *Coefficients* | | *Change statistics* | | *Coefficients* | | *Change statistics* | |
|  |  | Beta^c^ | p-val | R^2^ | *p(F)* | Beta^c^ | p-val | R^2^ | *p(F)* | Partial^d^ | p-val | R^2^ | *p(F)* |
| DLPFC(46)-sgACC(25) | | 0.54 | 0.02 | 0.29 | 0.02 |  | |  | | 0.18 | 0.5 | 0.35 | 0.04 |
| DLPFC(46)-aDMN(a24) | |  | |  | | 0.58 | 0.01 | 0.33 | 0.01 | 0.31 | 0.2 |  |  |
| R^2^ | adj. R^2^ | 0.29 | | 0.24 | | 0.33 | | 0.29 | | 0.35 | | 0.27 | |
| **Suppl. Table 1. No additivity DLPFC-sgACC and DLPFC-aDMN.**  ^a^ Stepwise method  ^b^ Enter method  ^c^ Standardized coefficient  ^d^ Partial correlation coefficients: independent contribution of each factor after adjusting by the influence of the second factor in Model Combined | | | | | | | | | | | | | |

| **Linear regression^a^** | | **Model 4^b^** | | | | **Model 5^c^** | | | | **Model 6^d^** | | | | **Model 7^e^** | | | | |
| --- | --- | --- | --- | --- | --- | --- | --- | --- | --- | --- | --- | --- | --- | --- | --- | --- | --- | --- |
|  |  | **Coefficients** | | **Change**  **statistics** | | **Coefficients** | | **Change**  **statistics** | | **Coefficients** | | **Change statistics** | | **Coefficients** | | **Change statistics** | | |
|  |  | Partial^f^ | p-val | R^2^ | p(F) | Partial^f^ | p-val | R^2^ | p(F) | Partial^f^ | p-val | R^2^ | p(F) | Partial^f^ | p-val | R^2^ | p(F) | |
|  | Intra-VIS(ventral) | -0.63 | 0.007 | 0.27 | 0.026 | -0.59 | 0.01 | 0.27 | 0.026 | -0.56 | 0.02 | 0.27 | 0.026 | -0.54 | 0.025 | 0.27 | 0.026 | |
| ***Step_2_*** | DLPFC(46)-aDMN(a24) | 0.67 | 0.003 | 0.32 | 0.003 |  | |  | |  |  |  |  |  |  |  | |  |
|  | intra-DMN(s32/10r) |  |  |  |  | -0.61 | 0.009 | 0.27 | 0.009 |  |  |  |  |  |  |  |  |  |
|  | DLPFC(p9-46v)-DMN(a24) |  |  |  |  |  | |  | | 0.55 | 0.02 | 0.22 | 0.021 |  |  |  |  |  |
|  | DLPFC(46)-sgACC(25) |  |  |  |  |  |  |  |  |  | |  | | 0.55 | 0.02 | 0.22 | | 0.022 |
| R^2^ | adj. R^2^ | 0.60 | | 0.54 | | 0.55 | | 0.49 | | 0.50 | | 0.43 | | 0.49 | | 0.43 | | |
| **Suppl. Table 2. Linear regression of other Models for prediction of ECT response.** | | | | | | | | | | | | | | | | | | |
| ^a^ Stepwise method. Step_1_: 1-factor; Step_2_: 2-factor  ^b^ Model 4: Intra-VIS(ventral) and DLPFC(46)-aDMN(a24) | | | | | | | | | | | | | | | | | | |
| ^c^ Model 5: Intra-VIS(ventral) and intra-aDMN(s32/10r) | | | | | | | | | | | | | | | | | | |
| ^d^ Model 6: Intra-VIS(ventral) and DLPFC(p9-46v)-aDMN(a24) | | | | | | | | | | | | | | | | | | |
| ^e^ Model 7: Intra-VIS(ventral) and DLPFC(46)-aDMN(25) | | | | | | | | | | | | | | | | | | |
| ^f^ Partial correlation coefficients: independent contribution of each factor after adjusting by the influence of the second factor in Models 4-7 | | | | | | | | | | | | | | | | | | |

| **Logistic regression^a^**  **Remission (HDRS-24≤7)** | | **Model 4^b^** | | **Model 5^c^** | | **Model 6^d^** | | **Model 7^e^** | |
| --- | --- | --- | --- | --- | --- | --- | --- | --- | --- |
|  |  | OR  per 1SD | p-val | OR  per 1SD | p-val | OR  per 1SD | p-val | OR  per 1SD | p-val |
| ***Step_1_*** | Intra-VIS(ventral) | 0.07 | 0.002 | 0.09 | 0.002 | 0.19 | 0.006 | 0.13 | 0.008 |
| ***Step_2_*** | DLPFC(46)-DMN(a24) | 28.81 | 0.023 |  |  |  |  |  |  |
|  | intra-DMN(s32/10r) |  |  | 0.06 | 0.004 |  |  |  |  |
|  | DLPFC(p9-46v)-DMN(a24) |  |  |  |  | 6.97 | 0.02 |  |  |
|  | DLPFC(46)-sgACC(25) |  |  |  |  |  |  | 4.89 | 0.021 |
| **% Accuracy** | | ***Step_1_*** | ***Step2*** | ***Step_1_*** | ***Step2*** | ***Step_1_*** | ***Step2*** | ***Step_1_*** | ***Step2*** |
| Global^f^ | | 77.8% | 94.4% | 77.8% | 100% | 77.8% | 94.4% | 77.8% | 88.9% |
| Non-remitter | | 77.8% | 88.9% | 77.8% | 100% | 77.8% | 100% | 77.8% | 88.9% |
| Remitter | | 77.8% | 100% | 77.8% | 100% | 77.8% | 88.9% | 77.8% | 88.9% |
| **Suppl. Table 3. Logistic regression of other Models for prediction of ECT response.**  ^a^ Stepwise method. Step_1_: 1-factor; Step_2_: 2-factor. Firth, D. (1993) Bias reduction of maximum likelihood estimates. *Biometrika* **80**, 27–3  ^b^ Model 4: Intra-VIS(ventral) and DLPFC(46)-aDMN(a24)  ^c^ Model 5: Intra-VIS(ventral) and intra-aDMN(s32/10r)  ^d^ Model 6: Intra-VIS(ventral) and DLPFC(p9-46v)-aDMN(a24)  ^e^ Model 7: Intra-VIS(ventral) and DLPFC(46)-aDMN(25)  ^f^ Average accuracy non-remitter and remitter; cut-point 0.5 | | | | | | | | | |

| **Change in connectivity** | | **Correlation analyses** | | | **Partial correlation analyses** | | | | | |  |
| --- | --- | --- | --- | --- | --- | --- | --- | --- | --- | --- | --- |
|  |  |  |  |  | **Adjusted by RelRMS** | | | **Adjusted by AbsRMS** | | |  |
|  |  | **r** | **uncorrected**  **p-value** | **corrected**  **p-value** | **r** | **uncorrected**  **p-value** | **corrected**  **p-value** | **r** | **uncorrected**  **p-value** | **corrected**  **p-value** |  |
| **Initial**  **DLPFC_neg_**  **analyses** | **DLPFC(46)-sgACC(25)** | **-0.626** | **0.005** | **0.022** | **-0.632** | **0.006** | **0.041** | **-0.641** | **0.006** | **0.022** |  |
|  | **DLPFC(46)-aDMN(a24)** | **-0.630** | **0.005** | **0.022** | **-0.603** | **0.010** | **0.041** | **-0.642** | **0.005** | **0.022** |  |
|  | **DLPFC(p9-46v)-aDMN(a24)** | **-0.577** | **0.012** | **0.032** | -0.546 | 0.023 | 0.063 | **-0.570** | **0.017** | **0.045** |  |
| **Subsequent network**  **analyses** | **DLPFC(46)-aDMN(s32)** | **-0.606** | **0.008** | **0.048** | -0.650 | 0.005 | 0.072 | -0.644 | 0.005 | 0.057 |  |
|  | **DLPFC(46)-aDMN(10r)** | **-0.597** | **0.009** | **0.049** | -0.595 | 0.012 | 0.072 | -0.612 | 0.009 | 0.057 |  |
|  | DLPFC(p9-46v)-aDMN(s32) | -0.519 | 0.027 | 0.080 | -0.496 | 0.043 | 0.125 | -0.517 | 0.034 | 0.098 |  |
|  | DLPFC(p9-46v)-aDMN(10r) | -0.546 | 0.019 | 0.066 | -0.578 | 0.015 | 0.072 | -0.578 | 0.015 | 0.066 |  |
|  | DLPFC(p9-46v)-pDMN(31pv) | -0.567 | 0.014 | 0.056 | -0.561 | 0.019 | 0.081 | -0.575 | 0.016 | 0.066 |  |
|  | DLPFC(p9-46v)-VIS(MT+) | 0.564 | 0.015 | 0.056 | 0.543 | 0.024 | 0.081 | 0.555 | 0.021 | 0.066 |  |
|  | pDMN(31pv)-VIS(MT+) | -0.534 | 0.023 | 0.072 | -0.487 | 0.048 | 0.129 | -0.511 | 0.036 | 0.098 |  |
|  | **pDMN(31pv)-VIS(dorsal)** | **-0.616** | **0.007** | **0.048** | -0.607 | 0.010 | 0.072 | -0.600 | 0.011 | 0.059 |  |
|  | **Intra-DLPFC(46)** | **0.646** | **0.004** | **0.048** | 0.586 | 0.013 | 0.072 | 0.632 | 0.006 | 0.057 |  |
|  | **Intra-DLPFC(p9-46v/46)** | **0.691** | **0.001** | **0.048** | 0.651 | 0.005 | 0.072 | 0.678 | 0.003 | 0.057 |  |
|  | **Intra-aDMN(10r)** | **0.626** | **0.005** | **0.048** | 0.624 | 0.007 | 0.072 | 0.626 | 0.007 | 0.057 |  |
|  | Intra-aDMN(s32/10r) | 0.585 | 0.011 | 0.051 | 0.539 | 0.026 | 0.081 | 0.565 | 0.018 | 0.066 |  |
|  | **Intra-pDMN(31pv/v23ab)** | **0.611** | **0.007** | **0.048** | 0.617 | 0.008 | 0.072 | 0.612 | 0.009 | 0.057 |  |
|  | Intra-VIS(ventral) | 0.455 | 0.058 | 0.147 | 0.352 | 0.166 | 0.315 | 0.422 | 0.092 | 0.205 |  |
| ***Follow-up VIS***  ***analyses*** | ***DLPFC(p9-46v)-MT+(LO1)*** | ***0.648*** | ***0.004*** | ***0.048*** | ***0.618*** | ***0.008*** | ***0.041*** | *0.650* | *0.005* | *0.052* |  |
|  | *DLPFC(p9-46v)-MT+(FST)* | *0.565* | *0.015* | *0.052* | *0.562* | *0.019* | *0.071* | *0.561* | *0.019* | *0.072* |  |
|  | *DLPFC(p9-46v)-MT+(V3CD)* | *0.476* | *0.046* | *0.080* | *0.493* | *0.044* | *0.095* | *0.507* | *0.038* | *0.095* |  |
|  | ***pDMN(31pv)-dorsal(V3A)*** | ***-0.592*** | ***0.010*** | ***0.048*** | ***-0.653*** | ***0.004*** | ***0.034*** | *-0.603* | *0.010* | *0.052* |  |
|  | ***pDMN(31pv)-dorsal(V3B)*** | ***-0.614*** | ***0.007*** | ***0.048*** | ***-0.699*** | ***0.002*** | ***0.027*** | *-0.628* | *0.007* | *0.052* |  |
|  | *pDMN(31pv)-dorsal(V6A)* | *-0.553* | *0.017* | *0.052* | *-0.499* | *0.041* | *0.095* | *-0.531* | *0.028* | *0.085* |  |
| **Suppl. Table 4. Change in connectivity measures and change in depression scores.** Initial DLPFC_neg_ analyses tested 8 correlations involving RSFC between DLPFC_neg_(46, p9-46v, a9-46v, 9-46d) and sgACC(25) or rostral ACC(a24) to choose significant (uncorrected p values) DLPFC_neg_ parcels for subsequent network analyses. Subsequent network analyses tested 38 correlations involving RSFC within and between DLPFC_neg_(46, p9-46v), DMN or VIS networks. Follow-up VIS analyses queried which parcels within MT+ (p=9) and dorsal (p=6) were driving the observed regional comparisons. These analyses used all parcels within each region and were therefore FDR corrected for 15 comparisons each. We include in Suppl. Table 4 all significant (uncorrected p values) connections from correlation analyses; those that survived multiple comparison correction controlling for FDR are highlighted. | | | | | | | | | | | |

| \| **Nomenclature** \| **Region** \| **Area name** \| \| --- \| --- \| --- \| \| DLPFC or DLPFC_neg_ \| Dorsolateral prefrontal cortex \| 46, p9-46v, a9-46v, 9-46d \| \| sgACC \| Subgenual part of anterior cingulate cortex \| 25 \| \| aDMN \| Anterior cingulate and medial prefrontal cortex \| a24, s32, 10r \| \| pDMN \| Posterior cingulate cortex \| 31pv, v23ab \| \| VIS(ventral) \| Ventral stream visual cortex \| V8, VVC, PIT, FFC, VMV1, VMV2, VMV3 \| \| VIS(dorsal) \| Dorsal stream visual cortex \| V3A, V3B, V6, V6A, V7, IPS1 \| \| VIS(MT+) \| MT+ complex and neighboring visual areas \| V3CD, LO1, LO2, LO3, V4t, FST, MT, MST, PH \| |
| --- | --- | --- | --- | --- | --- | --- | --- | --- | --- | --- | --- | --- | --- | --- | --- | --- | --- | --- | --- | --- | --- | --- | --- | --- |

**Suppl. Table 5: Cortical areas and regions used for analyses.** ROIs from the HCP’s multi-modal parcellation v1.0.

## Supplementary References

1 Redlich, R. *et al.* Prediction of Individual Response to Electroconvulsive Therapy via Machine Learning on Structural Magnetic Resonance Imaging Data. *JAMA Psychiatry* **73**, 557-564, doi:10.1001/jamapsychiatry.2016.0316 (2016).

2 Argyelan, M. *et al.* Subgenual cingulate cortical activity predicts the efficacy of electroconvulsive therapy. *Transl Psychiatry* **6**, e789, doi:10.1038/tp.2016.54 (2016).

3 Mayberg, H. S. *et al.* Regional metabolic effects of fluoxetine in major depression: serial changes and relationship to clinical response. *Biological psychiatry* **48**, 830-843 (2000).

4 Fox, M. D., Buckner, R. L., White, M. P., Greicius, M. D. & Pascual-Leone, A. Efficacy of transcranial magnetic stimulation targets for depression is related to intrinsic functional connectivity with the subgenual cingulate. *Biological psychiatry* **72**, 595-603, doi:10.1016/j.biopsych.2012.04.028 (2012).

5 DeRubeis, R. J., Siegle, G. J. & Hollon, S. D. Cognitive therapy versus medication for depression: treatment outcomes and neural mechanisms. *Nature reviews. Neuroscience* **9**, 788-796, doi:10.1038/nrn2345 (2008).

6 Mayberg, H. S. Modulating dysfunctional limbic-cortical circuits in depression: towards development of brain-based algorithms for diagnosis and optimised treatment. *British medical bulletin* **65**, 193-207 (2003).

7 Abbott, C. C. *et al.* Electroconvulsive therapy response in major depressive disorder: a pilot functional network connectivity resting state FMRI investigation. *Front Psychiatry* **4**, 10, doi:10.3389/fpsyt.2013.00010 (2013).

8 Beall, E. B. *et al.* Effects of electroconvulsive therapy on brain functional activation and connectivity in depression. *J ECT* **28**, 234-241, doi:10.1097/YCT.0b013e31825ebcc7 (2012).

9 Perrin, J. S. *et al.* Electroconvulsive therapy reduces frontal cortical connectivity in severe depressive disorder. *Proceedings of the National Academy of Sciences of the United States of America* **109**, 5464-5468, doi:10.1073/pnas.1117206109 (2012).

10 Cano, M. *et al.* Modulation of Limbic and Prefrontal Connectivity by Electroconvulsive Therapy in Treatment-resistant Depression: A Preliminary Study. *Brain Stimul* **9**, 65-71, doi:10.1016/j.brs.2015.08.016 (2016).

11 Philippi, C. L., Motzkin, J. C., Pujara, M. S. & Koenigs, M. Subclinical depression severity is associated with distinct patterns of functional connectivity for subregions of anterior cingulate cortex. *J Psychiatr Res* **71**, 103-111, doi:10.1016/j.jpsychires.2015.10.005 (2015).

12 Mayberg, H. S. *et al.* Reciprocal limbic-cortical function and negative mood: converging PET findings in depression and normal sadness. *Am J Psychiatry* **156**, 675-682, doi:10.1176/ajp.156.5.675 (1999).

13 Ongur, D. & Price, J. L. The organization of networks within the orbital and medial prefrontal cortex of rats, monkeys and humans. *Cerebral cortex* **10**, 206-219 (2000).

14 Ghashghaei, H. T., Hilgetag, C. C. & Barbas, H. Sequence of information processing for emotions based on the anatomic dialogue between prefrontal cortex and amygdala. *NeuroImage* **34**, 905-923, doi:10.1016/j.neuroimage.2006.09.046 (2007).

15 Glasser, M. F. *et al.* A multi-modal parcellation of human cerebral cortex. *Nature* **536**, 171-178, doi:10.1038/nature18933 (2016).

16 Wang, L., Hermens, D. F., Hickie, I. B. & Lagopoulos, J. A systematic review of resting-state functional-MRI studies in major depression. *J Affect Disord* **142**, 6-12, doi:10.1016/j.jad.2012.04.013 (2012).

17 Guo, W. *et al.* Functional and anatomical brain deficits in drug-naive major depressive disorder. *Prog Neuropsychopharmacol Biol Psychiatry* **54**, 1-6, doi:10.1016/j.pnpbp.2014.05.008 (2014).

18 Guo, W. *et al.* Abnormal default-mode network homogeneity in first-episode, drug-naive major depressive disorder. *PloS one* **9**, e91102, doi:10.1371/journal.pone.0091102 (2014).

19 Lai, C. H. & Wu, Y. T. Decreased inter-hemispheric connectivity in anterior sub-network of default mode network and cerebellum: significant findings in major depressive disorder. *Int J Neuropsychopharmacol* **17**, 1935-1942, doi:10.1017/S1461145714000947 (2014).

20 Peng, D. *et al.* Dissociated large-scale functional connectivity networks of the precuneus in medication-naive first-episode depression. *Psychiatry Res* **232**, 250-256, doi:10.1016/j.pscychresns.2015.03.003 (2015).

21 Zhu, X. *et al.* Evidence of a dissociation pattern in resting-state default mode network connectivity in first-episode, treatment-naive major depression patients. *Biological psychiatry* **71**, 611-617, doi:10.1016/j.biopsych.2011.10.035 (2012).

22 Mayberg, H. S. *et al.* Cingulate function in depression: a potential predictor of treatment response. *Neuroreport* **8**, 1057-1061 (1997).

23 Joel, S. E., Caffo, B. S., van Zijl, P. C. & Pekar, J. J. On the relationship between seed-based and ICA-based measures of functional connectivity. *Magn Reson Med* **66**, 644-657, doi:10.1002/mrm.22818 (2011).

24 van Waarde, J. A. *et al.* A functional MRI marker may predict the outcome of electroconvulsive therapy in severe and treatment-resistant depression. *Molecular psychiatry* **20**, 609-614, doi:10.1038/mp.2014.78 (2015).

25 Mulders, P. C. *et al.* Default mode network coherence in treatment-resistant major depressive disorder during electroconvulsive therapy. *J Affect Disord* **205**, 130-137, doi:10.1016/j.jad.2016.06.059 (2016).

26 Buckner, R. L., Andrews-Hanna, J. R. & Schacter, D. L. The brain's default network: anatomy, function, and relevance to disease. *Annals of the New York Academy of Sciences* **1124**, 1-38, doi:10.1196/annals.1440.011 (2008).

27 Raichle, M. E. *et al.* A default mode of brain function. *Proceedings of the National Academy of Sciences of the United States of America* **98**, 676-682, doi:10.1073/pnas.98.2.676 (2001).

28 Buckner, R. L. & Carroll, D. C. Self-projection and the brain. *Trends Cogn Sci* **11**, 49-57, doi:10.1016/j.tics.2006.11.004 (2007).

29 Fales, C. L. *et al.* Altered emotional interference processing in affective and cognitive-control brain circuitry in major depression. *Biol Psychiatry* **63**, 377-384, doi:10.1016/j.biopsych.2007.06.012 (2008).

30 Sheline, Y. I. *et al.* The default mode network and self-referential processes in depression. *Proceedings of the National Academy of Sciences of the United States of America* **106**, 1942-1947, doi:10.1073/pnas.0812686106 (2009).

31 Rose, E. J., Simonotto, E. & Ebmeier, K. P. Limbic over-activity in depression during preserved performance on the n-back task. *NeuroImage* **29**, 203-215, doi:10.1016/j.neuroimage.2005.07.002 (2006).

32 Vasic, N., Walter, H., Sambataro, F. & Wolf, R. C. Aberrant functional connectivity of dorsolateral prefrontal and cingulate networks in patients with major depression during working memory processing. *Psychological medicine* **39**, 977-987, doi:10.1017/S0033291708004443 (2009).

33 Wagner, G. *et al.* Cortical inefficiency in patients with unipolar depression: an event-related FMRI study with the Stroop task. *Biological psychiatry* **59**, 958-965, doi:10.1016/j.biopsych.2005.10.025 (2006).

34 Stern, E. R., Fitzgerald, K. D., Welsh, R. C., Abelson, J. L. & Taylor, S. F. Resting-state functional connectivity between fronto-parietal and default mode networks in obsessive-compulsive disorder. *PloS one* **7**, e36356, doi:10.1371/journal.pone.0036356 (2012).
